# Two New Isoprenoid Flavonoids from *Sophora flavescens* with Antioxidant and Cytotoxic Activities

**DOI:** 10.3390/molecules26237228

**Published:** 2021-11-29

**Authors:** Jingjing Li, Yan Lin, Lei He, Rongxiu Ou, Tao Chen, Xu Zhang, Qirui Li, Zhu Zeng, Qingde Long

**Affiliations:** 1 Guizhou Provincial Key Laboratory of Pharmaceutics, Guiyang 550025, China; lijingjing1231@126.com (J.L.); linyan@gmc.edu.cn (Y.L.); h18385617621@163.com (L.H.); ourongxiu2021@163.com (R.O.); CT142123@163.com (T.C.); xuzhanggmu2018@163.com (X.Z.); lqrnd2008@163.com (Q.L.); zengzhu@gmc.edu.cn (Z.Z.); 2 School of Pharmaceutical Sciences, Guizhou Medical University, Guiyang 550025, China; 3 State Key Laboratory of Functions and Applications of Medicinal Plants, Guizhou Medical University, Guiyang 550025, China; 4 Engineering Center of Cellular Immunotherapy of Guizhou Province, Guizhou Medical University, Guiyang 550025, China

**Keywords:** *Sophora flavescens*, isoprenoid flavonoids, antioxidant activity, cytotoxicity

## Abstract

*Sophora flavescens* is a regularly used traditional Chinese medicine. In an attempt to discover adequate active agents, the isoprenoid flavonoids from *S. flavescens* were further investigated. In this work, two new compounds (**1**–**2**, kurarinol A-B) together with 26 known ones (**3**–**28**) were isolated and elucidated on the basis of extensive NMR, UV and MS analyses. Furthermore, the antioxidant activity of all constituents was assessed through ABTS, PTIO and DPPH methodologies and also were evaluated for cytotoxic activity by three tumor cell lines (HepG2, A549 and MCF7) and one human normal cell line (LO2 cells). As a result, a multitude of components revealed significant inhibitory activity. In particular, compound **1**–**2** (kurarinol A-B), two new flavanonols derivatives, exhibited the most potent ABTS inhibitory activity with IC_50_ of 1.21 µg/mL and 1.81 µg/mL, respectively. Meanwhile, the new compound **1** demonstrated remarkable cytotoxicity against three cancer cells lines with IC_50_ values ranging from 7.50–10.55 μM but showed little effect on the normal cell. The two new isoprenoid flavonoids could be promising antioxidant and anti-tumor nature agents.

## 1. Introduction

*Sophora flavescens* (Ku-Shen in Chinese), derived from the Fabaceae family, is widely distributed in China as a herb or shrub, and has a strong bitter taste ([1], pp. 202–203). In 200 A.D., *S. flavescens* was recorded in the traditional Chinese medicine masterpiece *Shen Nong’s Herbal Classic* for the first time for treating inflammation, solid tumors, and other disorders [2]. It was also included in the Pharmacopoeia of the People’s Republic of China in 1977 for the treatment of dysentery, hematochezia, jaundice, oliguria, vulvar swelling, and ulcers [3]. In addition, in some other Asian countries, such as Korea and Japan, *S. flavescens* is known as a commonly herb for antipyretic, analgesic, antihelmintic and stomachic therapies [4,5,6].

According to reports, alkaloids and flavonoids were the main bioactive components of *S. flavescens*. To date, a total of more than 60 alkaloids and 120 flavonoids have been isolated from *S. flavescens* [7]. Among them, matrine and oxymatrine are the most frequently employed as quality control markers in China ([1], pp. 189–190). Recently, flavonoids from the flowers of *S. flavescens*, *polygonum cuspidatum*, *radix sophorae tonkinensis*, *herba ephedrae*, *salvia miltiorrhiza* and *astragali radix* have drawn worldwide attention for their potential health benefits [8]. The structures of flavonoids with a lavanduly, prenyl or 1,1-dimethylallyl group show noticeable biological activity and 55 isoprenoid flavonoids were reviewed from *S. flavescens* in our previous work, all of which played an important roles in antimicrobial, anti-inflammatory, antidiabetic and anti-tumor activities [9,10,11].

However, few studies have concentrated on isoprenoid flavonoids for antioxidant capacity and cytotoxicity. Several studies have shown that a mass of free radicals can be generated during metabolic processes in the human body [12]. These free radicals could lead to oxidative stress and homeostatic imbalance without treatment and removal in a timely manner, and could further bring about some chronic diseases such as diabetes, angiocardiopathy and cancer [13]. To address this point, we decided to explore the active substances from *S. flavescens*. In this work, **2** new and **26** known compounds were identified by combined spectroscopy. These compounds were tested for the inhibitory activities of cytotoxicity on human cancer cells (human liver cancer cell HepG2, human lung cancer cell A549 and human breast cancer MCF7) and human normal liver cells (LO2 cells), together with ABTS (2-2′-azinobis-3-ethylben-zthia zoline-6-sulphonate), PTIO (2-phenyl-4,4,5,5-tetramethylimidazoline-1-oxyl 3-oxide) and DPPH (1,1-diphenyl-2-picrylhydrazyl) free radical scavenging inhibition activities. To sum up, a new compound named kurarinol A (**1**) exhibited excellent cytotoxic and antioxidant activities.

## 2. Results and Discussion

### 2.1. Compounds Structure Elucidation

**Figure 1:**
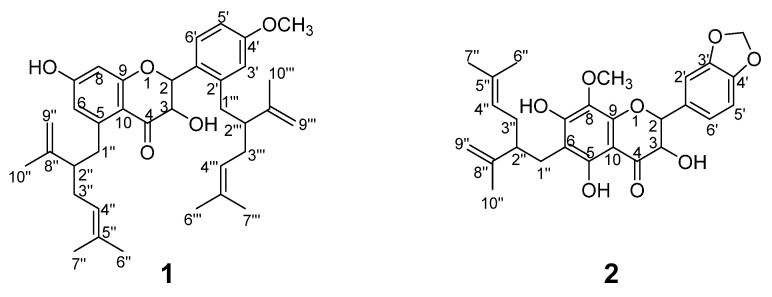
Structures of the compounds **1**–**2** from *S. flavescens*.

**Figure 2:**
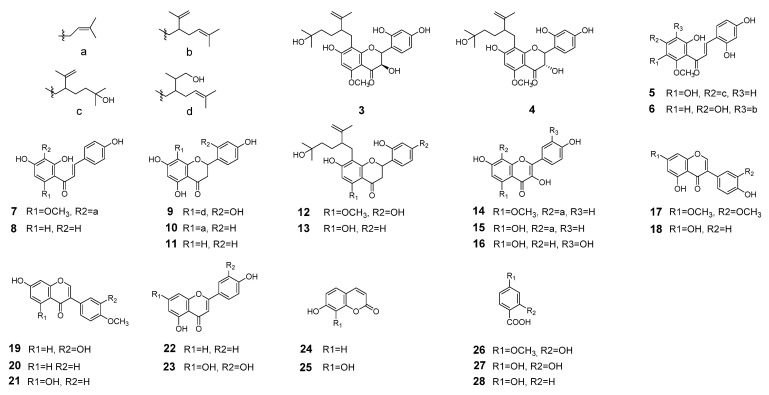
Structures of the compounds **3**–**28** from *S. flavescens*.

The ethyl acetate (EtOAc) extract of *S. flavescens* was repeatedly subjected to silica gel column, polyamide, sephadex LH-20 and further purified by semi-preparative RP-HPLC. Finally, a total of 28 pure compounds were obtained, including two new compounds (Figure 1) together with 26 know ones (Figure 2). These constituents belong to flavanonols (**1**–**4**), chalcones (**5**–**8**), flavanones (**9**–**13**), flavonols (**14**–**16**), isoflavones (**17**–**21**), flavones (**22**–**23**), and other structural types (**24**–**28**). The separation procedure was described in Support Information (Figure S1, Support Information). The structures of new compounds were confirmed by extensive spectroscopic analyses.

The 26 known compounds were identified by comparison of NMR spectral data with those published in the literature. They were kushenol H (**3**) [14], kushenol L (**4**) [15], kuraridinol (**5**) [16], kuraridine (**6**) [15], xanthohumol (**7**) [17], isoliquiritigenin (**8**) [18], kushenol Q (**9**) [14], sophoraflavanone B (**10**) [14], naringenin (**11**) [19], kurarinol (**12**) [14], kushenol A(**13**) [6], sophoflavescenol (**14**) [17], noranhyoicaritin (**15**) [16], quercetin (**16**) [20], 7,3′-di-*O*-methyl (**17**) [21], genistein (**18**) [20], calycosin (**19**) [20], formononetin (**20**) [22], biochanin A (**21**) [22], 5,4′-dihydroxyflavone (**22**) [23], luteolin (**23**) [24], 7-hydroxycoumarin (**24**) [25], 7,8-dihydroxycoumarin (**25**) [25], 4-methoxysalicylic acid (**26**) [26], *b*-resorcylic acid (**27**) [26] and 4-hydroxybenzoic acid (**28**) [26].

**Figure 3:**
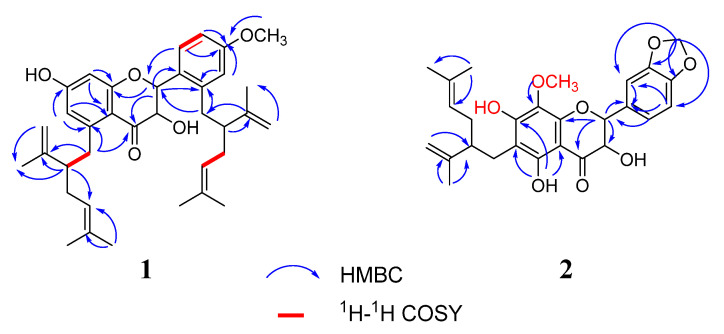
Key HMBC and H^1^-H^1^ COSY correlations for compounds **1**–**2**.

The HRESIMS data of compound **1** exhibited a [M − H]^−^ ion at *m*/*z* 557.1961 (calcd for C_36_H_45_O_5_, 557.1959), corresponding to a molecular formula of C_36_H_45_O_5_. The NMR spectrum mainly showed resonances for the characteristic signals at *δ_H_* 5.44 (1H, dd, *J* = 13.2 Hz, 2.4 Hz)/*δ_C_* 74.0 (C-2), 5.41 (1H, dd, *J* = 13.2 Hz, 2.4 Hz)/*δ_C_* 69.8 (C-3), and *δ_C_* 189.4 (C=O), suggesting that **1** had a flavanonol skeleton [14,27] (Table 1). The protons displaced two lavandulyl fragments signals at [(*δ_H_* 4.91, 1H, t/*δ_C_* 123.8, C-4″), (*δ_H_* 4.95, 1H, t, H-4‴), (*δ_H_* 4.56, br s, *δ_H_* 4.51, br s/*δ_C_* 117.0, C-9″), (*δ_H_* 4.48, br s, *δ_H_* 4.38, br s, H-9″)]. These two lavandulyl groups were located at C-5 and C-2′ positions according to the HMBC correlations of H-1″ (*δ_H_* 2.51) with C-5 (*δ_C_* 107.5), and H-1‴ *(δ_H_* 2.81) with C-2′ (*δ_C_* 116.8), respectively. Furthermore, the ABX-type protons were revealed at *δ_H_* 6.34 (1H, d, *J* = 1.2), 6.27 (1H, dd, *J* = 4.4, 8.4 Hz), 7.21 (1H, d, *J* = 8.4 Hz). The methoxy group was fused to C-4′ position according to the HBMC correlation of -OCH_3_ (*δ_H_* 3.70) with C-4′ (*δ_C_* 158.5). Two double peak protons at *δ_H_* 6.13 (1H, d, *J* = 2.4 Hz) and *δ_H_* 6.26 (1H, d, *J* = 2.4 Hz). The proton was located at C-6, which was confirmed by the HMBC correlations of H-6 (*δ_H_* 6.13) with two quaternary carbons at C-5 (*δ_C_* 107.5) and C-7(*δ_C_* 162.9) (Figure 3). Therefore, the planar structure of **1** was defined as 7-hydroxy-5,1 -bis (5-methyl-2-isopropyl-hexene) -flavanonol, and was named kurarinol A.

The molecular formula of **2** was determined to be C_27_H_30_O_8_ according to the HRESIMS *m*/*z* 481.1754 [M-H]^−^ (calcd for 481.1750). The ^1^H and ^13^C NMR spectra of **2** showed resonances at *δ_H_* 5.31 (1H, d, *J* = 10.8 Hz)/*δ_C_* 77.7 (C-2), 4.65 (1H, d, *J* = 10.8 Hz)/*δ_C_* 70.4 (C-3), and *δ_C_* 198.6 (C=O), indicating the presence of a flavanonol skeleton (Table 1). In addition, 1D and 2D NMR spectra (Figure 3) revealed a lavandulyl group (*δ_H_* 4.88, 1H, t), (*δ_H_* 4.44, br s, *δ_H_* 4.52, br s), (*δ_H_* 1,91, 2H, m), (*δ_H_* 1.45, 3H, s), (*δ_H_* 1.52, 6H, s), a methoxy moiety (*δ_H_* 3.58, *δ_C_* 55.3) and three hydroxyl groups (*δ_H_* 11.8, 9.47, 9.35), these data are similar to those of kushenol X [14]. Their slight changes were that the positions of the groups at C-6 and C-8 were interchangeable and C-8 from H turned into a methoxy group on ring A of **2**, as evidenced by the HMBC correlations from 7-OH (*δ_H_* 9.35) to C-8 (*δ_C_* 157.2), -OCH_3_ (*δ_H_* 3.58) to C-8 (*δ_C_* 157.2). Then, the lavandulyl group was located at C-6, supported by the key HMBC correlations of 5-OH (*δ_H_* 11.8) with C-6 (*δ_C_* 95.3). In ring B, the major differences were that 1,2,4-trisubstituted aromatic moiety evolved into 1,3,4-trisubstituted aromatic moiety, two hydroxyl groups lost a proton becoming a methylenedioxy ring and fused to C-3′ and C-4′. This deduction was confirmed by the HMBC correlations of -OCH_2_O- (*δ_H_* 5.95) with C-3′ (*δ_C_* 160.4), C-4′ (*δ_C_* 164.9), C-2′ (*δ_C_* 102.3) and C-5′ (*δ_C_* 106.4). Thus, compound **2** was identified as 5, 7-dihydroxy-8-methoxy-6-(5-methyl-2-iso-hexenyl)-3′,4′-methylene dioxy -flavanonol and was named kurarinol B.

### 2.2. Antioxidant Activities Research In Vitro

**Figure 4:**
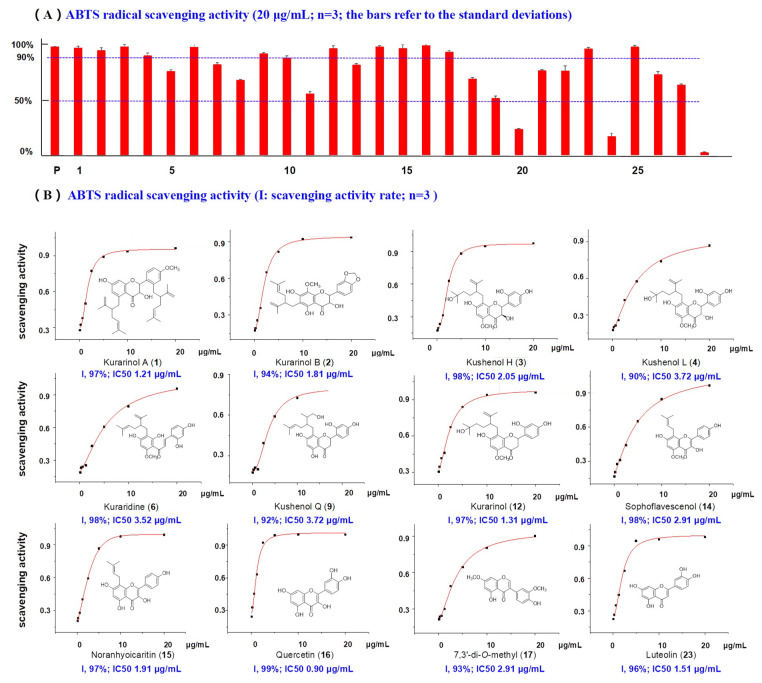
(**A**) ABTS radical scavenging activity of compounds **1**–**28** from *S. flavescens.* (p, positive control Vc, 20 μg/mL). (**B**) The concentration–scavenging activity curves for active compounds and their IC_50_ values.

The DPPH, ABTS and PTIO radicals can be used to evaluate antioxidant capacity of natural agents [28,29]. In this study, 28 compounds (20 μg/mL) from *S. flavescens* were estimated with vitamin C (VC) as the positive control. As shown in Figure 4, 12 compounds showed inhibition rates of >90% at 20 μg/mL, including **1**, **2**, **3**, **4**, **6**, **9**, **12**, **14**, **15**, **16**, **17** and **23**. Of these, all except kuraridine (**6**), are reported here for the first time [30]. In particular, new compounds **1** (kurarinol A), **2** (kurarinol B) and four known ones kurarinol (**12**), noranhyoicaritin (**15**), quercetin (**16**) and luteolin (**23**) displayed the most powerful activities, with IC_50_ values between 0.90 and 1.91 μg/mL. Furthermore, nine of the twelve flavonoids had one or more isoprenoid groups (lavandulyl, isoprenyl and 1,1-dimethylallyl groups). The isoprenoid group may be important for the ABTS radical scavenging activity.

**Figure 5:**
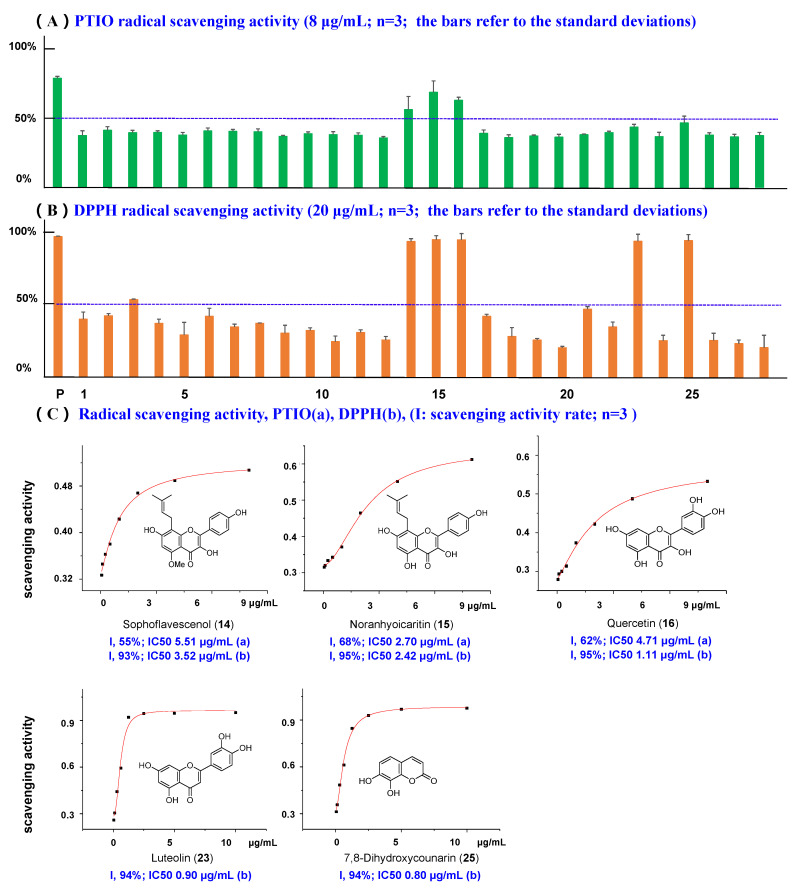
(**A**) PTIO radical scavenging activity of compounds **1**–**28**. (p, positive drug Vc, 8 μg/mL). (**B**) DPPH radical scavenging activity of compounds **1**–**28**. (p, positive drug Vc, 20 μg/mL). (**C**) The concentration–scavenging activity curves for active compounds and their IC_50_ values. (a: data of PTIO, b: data of DPPH).

Nowadays, PTIO radical scavenging activity is a universally recognized antioxidant screening method in vitro [31]. In this work, PTIO inhibitory activities of 28 compounds (8 µg/mL) isolated from *S. flavescens* were tested. VC was also used as a positive control. Among the 28 ingredients, only three compounds exhibited remarkable activities, namely sophoflavescenol (**14**), noranhyoicaritin (**15**), and quercetin (**16**) (Figure 5). Compounds **14** and **15** are reported here for the first time, with an IC_50_ of 5.51 µg/mL and 2.70 µg/mL, respectively. Compound **16** has previously been reported in [32].

Simultaneously, DPPH free radical scavenging method, as the most prevalent assay for evaluating antioxidant activity, was performed in this task and VC served as the positive control. According to the Figure 5 and Table S2 (Supporting Information), only five compounds (**14**, **15**, **16**, **23**, and **25**) inhibited DPPH activity by >90% at 20 µg/mL (the positive control VC showed an inhibition rate of 97% at 20 µg/mL). Compounds **16** (quercetin) and **23** (luteolin) have been published previously [33,34]. Compounds **14** (sophoflavescenol, IC_50_ 3.52 µg/mL), **15** (noranhyoicaritin, IC_50_ 2.42 µg/mL) and **25** (7,8-dihydroxycoumarin, IC_50_ 0.80 µg/mL) are reported here for the first time.

### 2.3. Cytotoxic Activities (HepG2, A549 and MCF7 Cancer Cells, LO2 Human Normal Cells)

**Figure 6:**
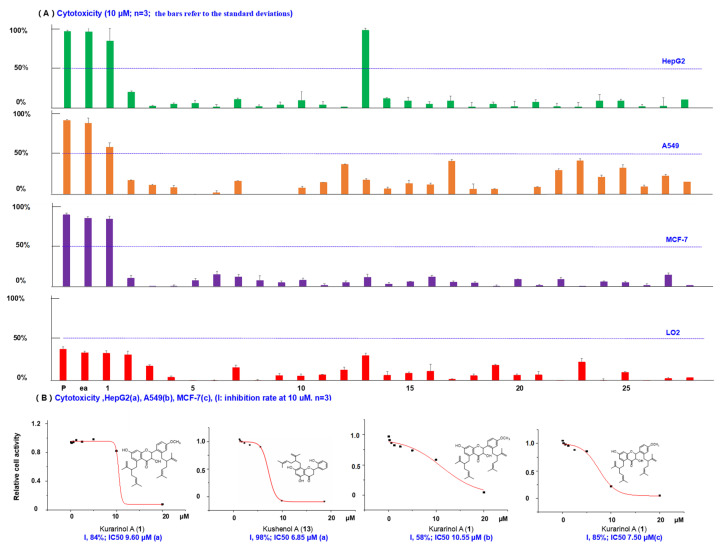
(**A**) Bioactivity screening results for cytotoxic inhibition activities of compounds **1**–**28**. (p, positive drug irinotecan, HepG2, 0.6 μM; A549, 1.0 μM; MCF-7, 0.9 μM; ea, EtOH crude extracts 25 μg/mL). (**B**) The dose–inhibition rate curves for active compounds and their IC_50_ values. (a: data of HepG2, b: data of A549, c: data of MCF-7).

Natural products with isoprenoid moieties are an important focus of research for scholars. Previous literatures reported that *S. flavescens* exhibited promising cytotoxicities against cancer cell lines [29,35]. In the work, cytotoxic activities of 28 compounds (10 μM) and the ethyl acetate (EtOAc) extract (25 μg/mL) were evaluated with three human cancer cell lines and one normal cell, with irinotecan as the positive control. IC_50_ values of 0.6 μM, 1.0 μM, and 0.9 μM for HepG2, A549, MCF7, respectively, were found (Figure 6). The results showed the EtOAc extract exhibited the strongest activity (73–96% inhibition against the three cancer cell lines at 25 μg/mL). Thus, we isolated the EtOAc extract to obtain 28 compounds, and tested their bioactivities.

As a result, kushenol A (**13**) displayed selectivity against different tumor cell lines and revealed IC_50_ values of 6.85 μM for HepG2 cells, could hardly inhibit MCF7 and A549 cells, and was discovered as cytotoxic agent for the first time. Interestingly, a new compound **1** (kurarinol A) demonstrated potent cytotoxicities against all the three human cancer cell lines with IC_50_ values in the range of 7.50–10.55 μM. The two lavandulyl groups might remarkably improve the cytotoxicity. Compounds **2**, **3** and **4** showed poor inhibitory activity against HepG2 cells, with inhibition rates of 19.9%, 1.9% and 4.6% at 10 μM. However, their isoprenoid derivative **1** inhibited the cells by 84.1%. Similar results were observed for compounds **2**, **3**, and **4** (10.3%, 4.5% and 0.7% inhibition against MCF7 cells) and their isoprenylated derivative, **1** (85.0% against MCF7 cells). We also tested the cytotoxicities of the above compounds against LO2 human normal cell lines (Figure 6). All of them showed weaker cytotoxic activity against the normal cells than the cancer cells.

## 3. Materials and Methods

### 3.1. Reagents and Materials

The extraction and separation of *S. flavescens* was performed using analytical or chromatographic grade organic solvents from Anhui Tiandi high-purity Solvent Co., Ltd. The ABTS, PTIO, DPPH, MTS and DMSO were obtained from Sigma-Aldrich (St. Louis, MO, USA). DMEM medium, the dual antibiotic mixture (penicillin–streptomycin), 0.25% Trypsin-EDTA, and FBS were purchased from Gibco Company. Phosphate Buffered Saline (PBS) was acquired from Shanghai Macklin Biochemical Co., Ltd. (Shanghai, China). Three human cancer cell lines (HepG2, A549 and MCF7 cells) were obtained from American Type Culture Collection (ATCC, Manassas, MD, USA) and human normal cells were acquired from The Key Laboratory of Optimal Utilization of Natural Medicine Resources (Guiyang, China). The positive control irinotecan was obtained from Shanghai Hongye Biotechnology Co., Ltd. (Shanghai, China).

### 3.2. General Experimental Procedures

UV spectra were measured on a Cary 300 Bio UV-visible spectrophotometer (Agilent Cary 300, Agilent Technologies Inc., Asia-Pacific, Mulgrave, Australia). IR spectra were recorded as KBr disks on a Nicolet NEXUS-470 FT-IR instrument (Thermo Nicolet NEXUS 470 FT-IR, Nicolet Inc., Madison, WI, USA). NMR spectra were recorded at 600 MHz for ^1^H NMR and 150 MHz for ^13^C NMR on a Bruker 600 M NEO (AVANCE 600 MHz, Brucker Technology Co., Ltd., Bruker, Germany) NMR spectrometer in DMSO-*d*_6_ with TMS used as the reference (unless otherwise stated). HRESIMS data were acquired on a Shimadzu UPHL-IT-TOF mass spectrometer (GC-2014, Shimadzu Enterprises Co., Ltd., Toyko, Japan). TLC was performed on precoated silica gel GF_254_ plates (Qingdao Marine Chemical Inc., Qingdao, China), and the spots were visualized under UV light (365 nm, 254 nm). Column chromatography was carried out using silica gel (100−200 mesh, Qingdao Marine Chemical Inc.), polyamide (100−200 mesh, Taizhou Luqiaosijia Plastic Factory, Taizhou, China), and Sephadex LH-20 (GE Healthcare Bio-Science AB, Uppsala, Sweden) as packing materials. Semi-preparative HPLC was performed on an Agilent 1200 instrument equipped with a YMC Pack ODS-A column (250 mm × 10 mm, i.d., 5 μm, YMC Co., Ltd., Kyoto, Japan).

### 3.3. Plant Material

The roots of *S. flavescens* were collected in September 2019 in Dafang county, Bijie city, Guizhou province, People’s Republic of China. The plant species of *S. flavescens* was confirmed by DNA barcoding analysis using the ITS sequences (Supplementary Information). Voucher specimen was deposited at the School of Pharmaceutical Sciences, Guizhou Medical University (Guiyang, China).

### 3.4. Extraction and Isolation

The dried power of *S. flavescens* (25 kg) was fully dipped in 95% and 75% EtOH for 7 days, three times, respectively. After concentrating the filtered liquor in a vacuum we obtained the extract. The extract was then dispersed in water and successively extracted with EtOAc and *n*-BuOH. The EtOAc extract (420 g) was separated on a silica gel column eluted with petroleum ether/ethyl acetate (1:0, 50:1, 10:1, 8:1, 6:1, 4:1, 2:1, 1:1, 0:1, *v*/*v*) to obtain fractions A-H. The eight fractions were separated by repeated column chromatography and preparative liquid chromatography to obtain the isoprenoid flavonoids **1**–**28**. The detailed separation procedure is described in Support Information (Support Information). Purities for all the compounds were above 95% by HPLC/UV analysis.

Kurarinol A (**1**): yellow powder; UV (MeOH) λmax 303 nm; IR (KBr) vmax 3438, 2971, 12933, 1602, 1502, 1452, 1415, 1284, 1099, 977 cm^−1^; ^1^H and ^13^C NMR data, see Table 1; HRESIMS *m*/*z* 557.1961 [M − H]**^−^** (calcd for C_36_H_45_O_5_, 557.1959).

Kurarinol B (**2**): yellow powder; UV (MeOH) λmax 295 nm; IR (KBr) vmax 3413, 2973, 2927, 1637, 1448, 1267, 1187, 1087, 977, 835 cm^−1^; ^1^H and ^13^C NMR data, see Table 1; HRESIMS *m*/*z* 481.1754 [M − H]**^−^** (calcd for C_27_H_29_O_8_, 481.1750).

### 3.5. DPPH Antioxidant Activity Assay

The in vitro antioxidant activity tests were conducted according to previous reports [30]. Among them, the DPPH free radical scavenging assay was slightly modified. Briefly, the DPPH material was precisely weighed, ethanol solution was added to obtain a concentration of 0.1 mmol/L (to use immediately, or store in the dark). Then, the compounds (100 µL, 40 µg/mL) with DPPH solution (100 µL) (A_i_) were added to 96-well plates and incubated for 30 min at room temperature. Subsequently, the activity was measured using the Thermo Scientific Varioskan LUX (Berthold, VL0L00D0, USA), with an absorbance of 570 nm. Vitamin C (VC) (40 µg/mL) was used as the positive control. The radical scavenging effect was calculated using the this equation: Free radical clearance = [1 − (A_i_ − A_j_)/A_0_] × 100% (the value of 100 µL ethanol with 100 µL DPPH solution as A_0_, the value of 100 µL compounds with 100 µL ethanol as A_j_).

### 3.6. PTIO Antioxidant Activity Assay

PTIO free radical scavenging was also performed following previous reports with some modification [28]. VC was used as the positive control. PTIO radicals were dissolved with phosphate buffer (PBS) (pH 7.4, 50 mM) with a concentration of 0.05 mg/mL. Then, the samples (40 µL, 40 µg/mL) and PTIO solution (160 µL) were added into 96-well plates (as the value of A_i_). After being thoroughly mixed, the reaction solution was incubated at 37 °C in a water-bath for 2 h. Then, the absorbance was measured at 557 nm. PTIO free radical scavenging was calculated using the above equation. (40 µL PBS with 160 µL PTIO as A_0_, 40 µL compounds with 160 µL PBS as A_j_).

### 3.7. ABTS Antioxidant Activity Assay

The third assay, the ABTS free radical scavenging screen was performed by previous experiments with slight changes [29]. First, we needed to prepare for the ABTS^+^ solution. The ABTS and potassium persulfate reagents were both dissolved in deionized water, to obtain concentrations of 7 mmol/L and 2.38 mmol/L, respectively. Then, they were mixed with equal volume at room temperature and kept away from light for 12 h, the mixed solution was diluted with ethanol until the absorbance of 0.70 ± 0.20. Thus, the ABTS^+^ solution was obtained. Furthermore, the compounds (100 µL, 40 µg/mL) with ABTS^+^ solution (100 µL) were also added to the 96-well plates (as the value of A_i_) and were interacted for 10 min at room temperature in the dark, then the absorbance at 734 nm was measured. VC was used as the positive control. Antioxidant capacity was calculated according to the first formula (the value of 100 µL ethanol with 100 µL ABTS^+^ solution as A_0_, the value of 100 µL compounds with 100 µL ethanol as A_j_). All experiments were carried out in triplicate.

### 3.8. Cytotoxic Activity Assay

The tests were conducted according to our previous reports [36,37]. The cells were grown in DMEM medium supplemented with 10% FBS, penicillin (100 U/mL), and streptomycin (100 μg/mL) in a 37 °C and 5% CO_2_ incubator. The cytotoxic activities were performed by the MTS assay. Briefly, 28 compounds were dissolved with DMSO. The cells were seeded at 1 × 10^5^ cells/mL in 96-well plates (100 µL/well) and cultured for 18 h when the cells filled the bottom of the wells. Then the supernatant was discard, the compounds (10 μM, 100 µL/well) were added to the culture and incubated for 24 h before cell viability measurement. 10 µL MTS reagent (0.5 mg/mL) were added and further incubated for 4 h. Finally, absorbance was read using an automatic micro-plate reader (Molecular Devices, USA) at 490 nm to test the cell viabilities, and the results were presented as the percent of non-treated control for each concentration. Irinotecan was used as the positive control. All measurements were repeated in triplicate. Data were expressed as the mean ± SD.

## 4. Conclusions

To sum up, a total of 28 compounds were isolated from the EtOAc extract of *S. flavescens* and two of them were new structures. They were identified mainly by NMR, UV and MS analyses. NMR spectroscopy was a powerful tool for the analysis of structure, the known frameworks mostly used 1D ^1^H-NMR spectrum, which simply showed signals for each of the hydrogen atoms in a compound. For the new ones, we also used 2D NMR spectrum, which revealed more signals between protons, adjacent ^13^C-^1^H and remote ^13^C-^1^H and was more useful and sensitive. The main contribution of UV spectrum in the structural identification of compounds was to determine the main skeleton. Kurarinol A-B were deduced to have flavonoid skeletons with maximum UV absorption at 303 and 295 nm, respectively. Additionally, the molecular weight of the compounds can be accurately determined through HRESIMS analyses. Furthermore, a number of isoprenoid flavonoids were found to be significant antioxidant inhibitors, and showed protective activities on ABTS, PTIO and DPPH free radical scavenging, including kurarinol A (**1**), kurarinol B (**2**), kushenol H (**3**), kushenol L (**4**), kuraridine (**6**), kushenol Q (**9**), kurarinol (**12**), sophoflavescenol (**14**), noranhyoicaritin (**15**), quercetin (**16**), 7,3′-di-O-methyl (**17**) and luteolin (**23**). Two new isoprenoid derivatives **1**–**2** (kurarinol A-B), exhibited the most potent antioxidant capacities against the ABTS enzyme with IC_50_ of 5.51 µg/mL and 2.70 µg/mL, respectively. Furthermore, the new compound **1** (kurarinol A) demonstrated significant cytotoxicity against the HepG2, A549 and MCF7 cell lines with IC_50_ values ranging from 7.50 to 10.55 μM. There were two lavandulyl groups which might remarkably improve the cytotoxicities. These compounds could be promising antioxidant and anti-tumor natural agents.

## Figures and Tables

**Table 1:** NMR data of compounds **1**–**2** (^1^H: 600 MHz, ^13^C: 150 MHz, DMSO-*d*_6_).

Position	1				2			
	*δ_c_*, Type		*δ_H_* (*J* in Hz)		*δ_C_*, Type		*δ_H_* (*J* in Hz)	
1								
2	74.0	CH	5.44, dd (2.4, 13.2)		77.7	CH	5.31, d (10.8)	
3	69.8	CH	5.41, dd (2.4, 13.2)		70.4	CH	4.65, d (10.8)	
4	189.4	C			198.6	C		
5	107.5	C						
6	92.6	CH	6.13, d (2.4)		95.3	C		
7	162.9	C			160.9	C		
8	106.8	CH	6.26, t		157.2	C		
9	155.6	C			158.5	C		
10	104.8	C			100.4	C		
1′	111.2	C			113.9	C		
2′	116.8	C			102.3	CH	6.33,d (1.8)	
3′	102.8	CH	6.34, d (1.2)		160.4	C		
4′	158.5	C			164.9	C		
5′	106.8	CH	6.27, dd (4.4, 8.4)		106.4	CH	6.25, dd (1.8, 8.4)	
6′	127.1	CH	7.21, d(8.4)		129.4	CH	7.15, d (8.4)	
1″			2.51, m		26.4	CH2	2.38, m	
2″	46.8	CH	2.41, m		46.3	CH	2.38, m	
3″	31.2	CH2	1.96, m		30.9	CH2		
4″	123.9	CH	4.91, t		123.4	CH	4.88, t	
5″	131.1	C			130.6	C		
6″	18.7	CH3	1.43, S		25.5	CH3	1.52, S	
7″	26.0	CH3	1.55, S		17.6	CH3	1.45, S	
8″	148.1	C			147.7	C		
9″	117.0	CH2	4.51, 4.56, br s		110.8	CH2	4.44, 4.52, br s	
10″	18.0	CH3	1.52, S		18.4	CH3	1.52, S	
1‴			2.81, m					
2‴	47.2	CH	2.41, m					
3‴	31.5	CH2	1.96, m					
4‴			4.95, t					
5‴								
6‴	18.0	CH3	1.49, S					
7‴	27.4	CH3	1.58, S					
8‴	148.4	C						
9‴			4.48, 4.38, br s					
10‴	25.9	CH3	1.51, S					
OCH3	55.7	CH3	3.70, S		55.3	CH3	3.36, S	
OH							11.8, S	
OH							9.47, S	
OH							9.35, S	
OCH2O							5.95, 5.65, S	

## Supplementary Materials

The Supplementary Materials are available on online. Detailed experimental procedures for the isolation of **1**–**28**, spectroscopic data (NMR, MS, UV and IR) for new compounds **1**–**2**, antioxidant activities of ABTS, PTIO and ABTS as well as cytotoxicities for HepG2, A549 and MCF-7 cancer cells, cytotoxicity and for LO2 human normal cells (PDF).

## Sample Availability

Samples of the compounds are available from the authors.
