# Supplementary material for: Two New Isoprenoid Flavonoids from Sophora flavescens with Antioxidant and Cytotoxic Activities
Source: Molecules. 2021 Nov 29;26(23):7228. doi: 10.3390/molecules26237228 (PMC8658773; doi:10.3390/molecules26237228)
Supplement: Supplementary file 1 [file molecules-26-07228-s001.zip › molecules-1448688-supplementary.pdf]

Supplementary Materials: Two New Isoprenoid Flavonoids from *Sophora Flavescens* with Antioxidant and Cytotoxic Activities

Jingjing Li, Yan Lin, Lei He, Rongxiu Ou, Tao Chen, Xu Zhang, Qirui Li, Zhu Zeng and Qingde Long

1. Isolation and Structural Characterization of *Sophora flavescens* Compounds.

The dried plant material (25 kg) was powdered and dipped in 95% and 75% EtOH for 7 days, three times, respectively. The filtered liquor was concentrated in vacuum to obtain the extract. Then the extract was dispersed in water successively using a quadruple volume of EtOAc and water-saturated n-BuOH to extract, three times for each solvent. The EtOAc extract (420 g) were further separated on a column of silica gel in 100-200 mesh with a petroleum ether/ethyl acetate gradient (v/v = 1:0, 50:1, 10:1, 8:1, 6:1, 4:1, 2:1, 1:1, 0:1) to obtain fractions A-H. The fractions were separated by repeated column chromatography and preparative liquid chromatography to obtain the alkaloid compounds **1–28**.

Fraction B was separated to silica gel column eluted with petroleum ether/EtOAc (20:1, 10:1, 8:1, 6:1, 4:1, 2:1, 1:1, 0:1, Pure methanol, v/v) to obtain fractions B-1, B-2 and B3. B-1 and B-2 were subjected to a Sephadex LH-20 column eluted with MeOH and were purified by semi-preparative HPLC to obtain two compounds and one compound, respectively, isoliquiritigenin (**8**, 18 mg), noranhyoicaritin (**15**, 112 mg) and 4-methoxysalicylic acid (**26**, 5mg). Fraction B-3 was subjected multiple times to the silica gel column, where some crystallization/precipitation appeared, to gain one compound, sophoraflavanone B (**10**, 35mg).

Fraction C was separated by silica gel column eluted with petroleum ether/EtOAc (10:1, 8:1, 6:1, 4:1, 2:1, 1:1, 0:1, methanol,v/v) and subjected to chromatography to obtain fraction C-1. Fraction C was further separated on the silica column eluted with dichloromethane/methanol (50:1, 40:1, 20:1, 10:1, 8:1, 6:1, 4:1, 2:1, 1:1, 0:1) and eluted with EtOAc/methanol (10:1, 6:1, 4:1, 2:1, 0:1), then the compounds were purified by semi-preparative HPLC to obtain kuraridinol (**5**, 2 mg), kushenol Q (**9**, 6 mg).

Fraction D was pale yellow and easily soluble in methanol and subjected to the Sephadex LH-20 column eluted with MeOH, repeated several times and was purified by semi-preparative HPLC to get two compounds, kushenol A (**13**, 40 mg) and sophoflavescenol (**14**, 21 mg).

Fraction E was separated by silica gel column eluted with dichloromethane/ methanol (50:1, 40:1, 20:1, 10:1, 8:1, 6:1, 4:1, 2:1, 1:1, 0:1, v/v) to obtain fractions E-1 and E-2, fraction E-1 was further separated by polyamide column with dichloromethane/methanol (30:1, 20:1, 10:1, 8:1, 6:1, 4:1, 2:1, 1:1, 0:1, v/v) once again and purified by Sephadex LH-20 column eluted with MeOH, and finally was purified by semi-preparative HPLC to obtain two compounds kurarinol B (**2**, 13 mg) and 4-hydroxybenzoic acid (**28**, 138 mg). At the same time, fraction E-2 was also separated by polyamide column with dichloromethane/methanol (30:1, 20:1, 10:1, 8:1, 6:1, 4:1, 2:1, 1:1, 0:1, v/v), then further separated by the silica gel column eluted with acetone/methanol (10:1, 8:1, 6:1, 4:1, 2:1, 1:1, 0:1, v/v) and purified by the Sephadex LH-20 column eluted with MeOH, and finally, purified by semi-preparative HPLC to acquire one compound, kurarinol A (**1**, 33 mg).

Fraction F was separated by silica gel column eluted with petroleum ether/EtOAc (20:1, 10:1, 8:1, 6:1, 4:1, 2:1, 1:1, 0:1, Pure methanol, v/v) to obtain fractions F-1 and F-2, fraction F-1 was once again separated by silica gel column eluted with dichloromethane/methanol (20:1, 10:1, 8:1, 6:1, 4:1, 2:1, 1:1, 0:1, v/v), next, purified by Sephadex LH-20 column eluted with MeOH and semi-preparative HPLC to acquire one compound, kuraridine (**6**, 8 mg). Fraction F-2 was separated by polyamide column eluted with dichloromethane/methanol (20:1, 10:1, 8:1, 6:1, 4:1, 2:1, 1:1, 0:1, v/v), then once again and purified by the Sephadex LH-20 column eluted with MeOH, and finally, purified by semi-preparative HPLC to obtain three compounds, kushenol H (**3**, 3 mg), kushenol L (**4**, 4mg) and kurarinol (**12**, 10 mg).

Fraction G was separated by silica gel column eluted with dichloromethane/methanol (20:1, 10:1, 8:1, 6:1, 4:1, 2:1, 1:1, 0:1, v/v) to obtain fractions G-1 and G-2, fraction G-1 was further separated by polyamide column with dichloromethane/methanol (50;1, 40:1, 30:1, 20:1, 10:1, 8:1, 6:1, 4:1, 2:1, 1:1, 0:1, v/v), then separated by silica gel column with dichloromethane/methanol or petroleum ether/EtOAc, and finally, purified by semi-preparative HPLC to obtain two compounds, xanthohumol (**7**, 15 mg) and naringenin (**11**, 14 mg). Meanwhile, fraction G-2 used the same steps for separation to gain three compounds, genistein (**18**, 31 mg), 7-hydroxycoumarin (**24**, 15 mg) and 7,8-dihydroxycoumarin (**25**, 16 mg).

Fraction H was separated by silica gel column eluted with petroleum ether/EtOAc (10:1, 8:1, 6:1, 4:1, 2:1, 1:1, 0:1, Pure methanol, v/v) to obtain fractions H-1, H-2 and H-3. Fraction H-1 was separated by silica gel column eluted with petroleum ether/EtOAc (6:1, 4:1, 2:1, 1:1, 0:1, Pure methanol, v/v), further purified by the Sephadex LH-20 column eluted with MeOH and semi-preparative HPLC to acquire two compounds, quercetin (**16**, 16 mg) and *b*-resorcylic acid (**27**, 5 mg). Fraction H-2 was separated by silica gel column, polyamide column and silica gel column, in turn, and further purified by the Sephadex LH-20 column eluted with MeOH and semi-preparative HPLC to acquire three compounds 7,3'-di-O-methyl (**17**, 3 mg), calycosin (**19**, 1 mg) and formononetin (**20**, 2 mg). Fraction H-3 was further purified by silica gel column, polyamide column, Sephadex LH-20 and semi-preparative HPLC to obtain three compounds, biochanin A (**21**, 32 mg), 5,4'-dihydroxyflavone (**22**, 3 mg) and luteolin (**23**, 20 mg).

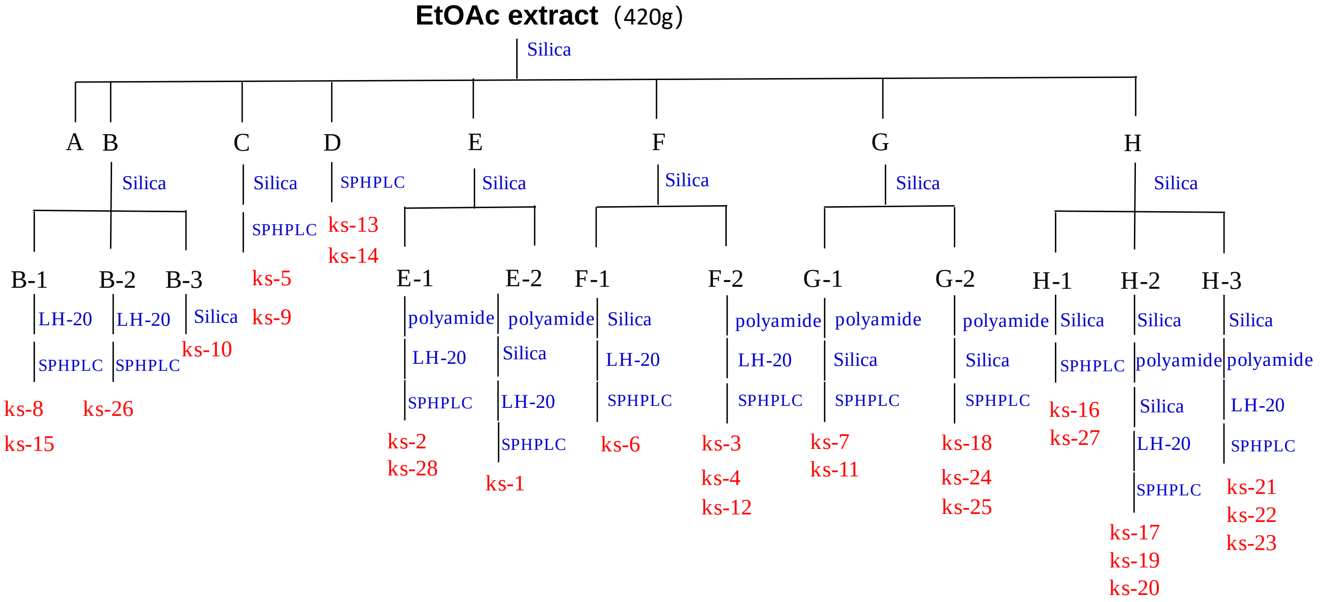

Figure S1. Flow chart for the isolation of compounds 1-28.

kushenol H (3): 1H-NMR (DMSO-d6, 600 MHz)， 5.19 (1H, d, J = 10.8 Hz, H-2), 4.33 (1H, d, J = 11.2 Hz, H-3), 6.12 (1H, s, H-6), 6.31 (1H, d, J = 2.4 Hz, H-3'), 6.21 (1H, dd, J = 2.4, 8.4 Hz, H-5'), 7.10 (1H, d, J = 8.4 Hz, H-6'), 2.29 (1H, m, H-2''), 1.25 (2H, m, H-3''), 2.41 (2H, m, H-4''), 0.97 (3H, s, H-6''), 0.97 (3H, s, H-7''), 4.50, 4.41 (each 1H, br s, H-9''), 1.49 (3H, s, H-10''), 3.71 (3H, s, -OCH3). 13C-NMR (DMSO-d6, 150 MHz), 76.9 (C-2), 68.7 (C-3), 190.9 (C-4), 159.4 (C-5), 92.3 (C-6), 162.4 (C-7), 106.9 (C-8), 161.9 (C-9), 114.1 (C-1'), 158.3 (C-2'), 102.3 (C-3'), 156.9 (C-4'), 106.1 (C-5'), 129.2 (C-6'), 20.7 (C-1''), 46.7 (C-2''), 29.0 (C-3''), 41.5 (C-4''), 71.4 (C-5''), 17.9 (C-6''), 147.9 (C-8''), 110.9 (C-9''), 55.3 (-OCH3). [1]

kushenol L (4): 1H-NMR (DMSO-d6, 600 MHz), 5.35 (1H, d, J = 1.6 Hz, H-2), 3.78 (1H, d, J = 2.0 Hz, H-3), 6.15 (1H, s, H-6), 6.31 (1H, d, J = 2.4 Hz, H-3'), 6.23 (1H, dd, J = 2.4, 8.4 Hz, H-5''), 7.29 (1H, d, J = 8.4 Hz, H-6'), 0.93 (3H, s, H-7''), 0.93 (3H, s, H-8''), 4.58, 4.53 (each 1H, br s, H-9''), 1.60 (3H, s, H-10''), 3.70 (3H, s, -OCH3). 13C-NMR (DMSO-d6, 150 MHz), 76.4 (C-2), 68.7 (C-3), 189.3 (C-4), 154.8 (C-5), 92.5 (C-6), 161.8 (C-7), 106.8 (C-8), 160.1 (C-9), 102 (C-10), 113.8 (C-1'), 157.8 (C-2'), 102.3 (C-3'), 105.8 (C-5'), 129.2 (C-6'), 46.5 (C-2''), 29.0 (C-3''), 41.4 (C-4''), 71.2 (C-5''), 18.2 (C-6''), 148.4 (C-8''), 29.4 (C-10''), 55.3 (-OCH3). [2]

kuraridinol (5): 1H-NMR (DMSO-d6, 600 MHz), 7.94 (1H, d, J = 15.6 Hz, H-1), 7.86 (1H, d, J = 15.6 Hz, H-2), 6.05 (1H, s, H-6), 14.86 (1H, s, 9-OH), 6.37 (1H, d, J = 2.4 Hz, H-3'), 6.32 (1H, dd, J = 2.4, 8.4 Hz, H-5'), 7.42 (1H, d, J = 8.4 Hz, H-6'), 2.55 (2H, m, H-1''), 2.38 (1H, m, H-2''), 1.02 (3H, s, H-6''), 1.09 (3H, s, H-7''), 4.56, 4.47 (each 1H, br s, H-9''), 1.64 (3H, s, H-10''), 3.81 (3H, s, -OCH3). [3]

kuraridine (6): 1H-NMR (DMSO-d6, 600 MHz), 7.84 (2H, q, H-1, H-2), 6.05 (1H, s, H-6), 14.84 (1H, s, H-9), 6.38 (1H, d, J = 3.6 Hz, H-3'), 6.30 (1H, dd, J = 3.6, 13.2 Hz, H-5'), 7.41 (1H, d, J = 13.2 Hz, H-6'), 1.01 (3H, s, H-6''), 1.01 (3H, s, H-7''), 4.57, 4.48 (each 1H, br s, H-9''), 1.64 (3H, s, H-10''), 3.83 (3H, s, -OCH3). 13C-NMR (DMSO-d6, 150 MHz), 138.7 (C-1), 191.9 (C-3), 161.3 (C-5), 102.7 (C-6), 165.3 (C-7), 106.8 (C-8), 159.2 (C-9), 113.8 (C-1'), 160.4 (C-2'), 108.1 (C-3'), 161.3 (C-4'), 102.7 (C-5'), 122.8 (C-6'), 29.1 (C-1''), 48.6 (C-2''), 29.6 (C-3''), 130.4 (C-5''), 27.2 (C-6''), 17.9 (C-7''), 148.1 (C-8''), 111.0 (C-9''), 26.7 (C-10''), 55.5 (-OCH3). [2]

xanthohumol (7): 1H-NMR (DMSO-d6, 600 MHz), 7.74 (1H, d, J = 15.0 Hz, H-1), 7.68 (1H, d, J = 15.0 Hz, H-2), 6.08

(1H, s, H-6), 10.56 (1H, s, 7-OH), 14.84 (1H, s, 9-OH), 10.0 (1H, s, 4'-OH), 7.58 (2H, d, J = 9.0 Hz, H-2', H-6'), 6.84 (2H, d, J = 9.0 Hz, H-3', H-5'), 3.14 (2H, d, J = 6.8 Hz, H-1''), 5.13 (1H, t, H-2''), 1.70 (3H, s, H-4''), 1.60 (3H, s, H-5''). 13C-NMR (DMSO-d6, 150 MHz), 143.1 (C-1), 126.5 (C-2), 192.1 (C-3), 105.0 (C-4), 165.1 (C-5), 91.4 (C-6), 162.9 (C-7), 107.8 (C-8), 161.0 (C-9), 124.2 (C-1'), 131.0 (C-2', C-6'), 108.1 (C-3', C-5'), 160.4 (C-4'), 21.8 (C-1''), 123.5 (C-2''), 26.0 (C-4''), 18.1 (C-5''). [4]

isoliquiritigenin (8): 1H-NMR (DMSO-d6, 600 MHz), 7.76 (1H, d, J = 15.6 Hz, H-1), 7.74 (1H, d, J = 15.6 Hz, H-2), 8.17 (1H, d, J = 13.2 Hz, H-5), 6.41 (1H, dd, J = 13.2, 3.6 Hz, H-6), 6.27 (1H, d, J = 3 Hz, H-8), 7.74 (2H, d, J = 10.0 Hz, H-2',H-6'), 6.84 (2H, d, J = 10.0 Hz, H-3', H-5'). 13C-NMR (DMSO-d6, 150 MHz), 145.6 (C-1), 120.5 (C-2), 190.5 (C-3), 131.2 (C-4), 109.2 (C-5), 102.5 (C-6), 158.8 (C-7), 113.9 (C-8), 165.9 (C-9), 126.5 (C-1'), 133.5 (C-2', C-6'), 116.8 (C-3', C-5'), 160.9 (C-4'). [5]

kushenol Q (9): 1H-NMR (DMSO-d6, 600 MHz), 5.58 (1H, dd, J = 2.8, 12.8 Hz, H-2), 2.68 (1H, d, J = 12.8 Hz, H-3α), 2.99 (1H, d, J = 12.8 Hz, H-3β), 5.90 (1H, s, H-6), 6.89 (1H, m, H-3'), 7.18 (1H, m, H-5'), 7.44 (1H, d, J = 7.2 Hz, H-6'), 2.70 (2H, m, H-1''), 5.12 (1H, t, H-4''), 1.58 (3H, s, H-6''), 1.56 (3H, s, H-7''), 3.06 (2H, m, H-9''), 1.25 (3H, s, H-10''), 12.16 (1H, s, 5-OH). 13C-NMR (DMSO-d6, 150 MHz), 74.1 (C-2), 196.5 (C-4), 159.9 (C-5), 95.7 (C-6), 161.4 (C-7), 107.3 (C-8), 115.6 (C-10), 126.8 (C-1'), 123.0 (C-3'), 154.5 (C-4'), 129.3 (C-6'), 125.5 (C-4''), 130.4 (C-5''), 25.8 (C-6''), 17.8 (C-7''), 41.2 (C-9''), 21.6 (C-10''). [1]

sophoraflavanone B (10): 1H-NMR (DMSO-d6, 600 MHz), 5.34 (1H, m, H-2), 3.08 (2H, m, H-3α, H-3β), 5.86 (1H, s, H-6), 7.31 (2H, d, J = 7.8 Hz, H-2',H-6'), 6.80 (2H, d, J = 8.4 Hz, H-3', H-5'), 5.12 (1H, t, H-2''), 1.58 (3H, s, H-4''), 1.55 (3H, s, H-5''). 13C-NMR (DMSO-d6, 150 MHz), 77.9 (C-2), 48.7 (C-3), 195.1 (C-4), 161.5 (C-5), 96.0 (C-6), 168.5 (C-7), 100.5 (C-8), 159.3 (C-9), 107.5 (C-10), 128.0 (C-2', C-6'), 115 (C-3',C-5'), 157.6 (C-4'), 21.5 (C-1''), 123.5 (C-2''), 129.6 (C-3''), 25.9 (C-4''), 17.7 (C-5''). [1]

naringenin (11): 1H-NMR (DMSO-d6, 600 MHz), 5.44 (1H, dd, J = 2.4, 12.6 Hz, H-2), 2.69 (1H, dd, J = 3.0, 16.8 Hz, H-3α), 3.28 (1H, m, H-3β), 5.81 (1H, s, H-6), 5.81 (1H, s, H-8), 7.31 (2H, d, J = 8.4 Hz, H-2',H-6'), 6.79 (2H, d, J = 8.4 Hz, H-3', H-5'). 13C-NMR (DMSO-d6, 150 MHz), 78.4 (C-2), 42.0 (C-3), 196.4 (C-4), 163.5 (C-5), 95.8 (C-6), 166.6 (C-7), 95.0 (C-8), 162.9 (C-9), 101.8 (C-10), 128.3 (C-2', C-6'), 115.2 (C-3',C-5'), 157.7 (C-4'). [6]

kurarinol (12): 1H-NMR (DMSO-d6, 600 MHz), 5.39 (1H, dd, J = 2.4, 12.8 Hz, H-2), 2.47 (1H, m, H-3α), 2.76 (1H, dd, J = 13.2, 16.4 Hz, H-3β), 6.13 (1H, s, H-6), 6.27 (1H, d, J = 2.0 Hz, H-3'), 6.24 (1H, dd, J = 2.0, 8.4 Hz, H-5'), 7.20 (1H, d, J = 8.4 Hz, H-6'), 1.26 (2H, m, H-3''), 2.30 (1H, m, H-4''), 0.95 (3H, s, H-6''), 0.95 (3H, s, H-7''), 4.56, 4.48 (each 1H, br s, H-9''), 1.56 (3H, s, H-10''). 13C-NMR (DMSO-d6, 150 MHz), 74.0 (C-2), 44.8 (C-3), 189.5 (C-4), 160.0 (C-5), 92.9 (C-6), 162.9 (C-7), 102.4 (C-8), 162.7 (C-9), 116.8 (C-10), 127.3 (C-1'), 155.7 (C-2'), 104.7 (C-3'), 158.6 (C-4'), 106.7 (C-5'), 107.5 (C-6'), 27.9 (C-1''), 47.1 (C-2''), 29.4 (C-3''), 42.0 (C-4''), 69.2 (C-5''), 29.9 (C-6''), 18.5 (C-7''), 148.6 (C-8''), 111.3 (C-9''), 26.9 (C-10''). [1]

kushenol A (13): 1H-NMR (DMSO-d6, 600 MHz), 5.63 (1H, dd, J = 3.0, 13.2 Hz, H-2), 2.77 (1H, dd, J = 2.4, 16.8 Hz, H-3α), 3.03 (1H, m, H-3β), 5.98 (1H, s, H-6), 6.89 (1H, d, J = 7.2 Hz, H-3'), 7.18 (1H, m, H-4'), 6.89 (1H, d, J = 7.2 Hz, H-5'), 7.49 (1H, dd, J = 1.2, 7.8 Hz, H-6'), 2.41 (1H, m, H-2''), 1.96 (2H, m, H-3''), 4.91 (1H, t, H-4''), 1.51 (3H, s, H-6''), 1.42 (3H, s, H-7''), 4.58, 4.51 (each 1H, br s, H-9''), 1.59 (3H, s, H-10''), 12.09 (1H, s, 5-OH) 13C-NMR (DMSO-d6, 150 MHz), 74.0

(C-2), 41.4 (C-3), 196.7 (C-4), 161.3 (C-5), 95.4 (C-6), 165.0 (C-7), 106.6 (C-8), 160.5 (C-9), 154.0 (C-2'), 115.3 (C-3'), 130.7 (C-4'), 119.0 (C-5'), 126.4 (C-6'), 30.7 (C-1''), 46.4 (C-2''), 26.6 (C-3''), 110.8 (C-4''), 125.3 (C-5''), 25.5 (C-6''), 17.6 (C-7''), 147.8 (C-8''), 18.7 (C-10''). [7]

sophoflavescenol (**14**): 1H-NMR (DMSO-d6, 600 MHz), 6.29 (1H, s, H-6), 8.03 (2H, d, J = 7.8 Hz, H-2', H-6'), 6.93 (2H, d, J = 8.4 Hz, H-3', H-5'), 3.50 (2H, d, J = 6.8 Hz, H-1''), 5.18 (1H, t, H-2''), 1.74 (3H, s, H-4''), 1.62 (3H, s, H-5''), 3.42 (3H, s, -OCH3), 12.30 (1H, s, 5-OH). 13C-NMR (DMSO-d6, 150 MHz), 146.7 (C-2), 135.6 (C-3), 176.2 (C-4), 158.3 (C-5), 97.9 (C-6), 161.4 (C-7), 105.6 (C-8), 159.2 (C-9), 115.5 (C-10), 129.4 (C-1'), 122.7 (C-2',C-6'), 102.9 (C-3', C-5'), 153.5 (C-4'), 21.3 (C-1''), 122.0 (C-2''), 131.0 (C-3''), 25.5 (C-4''), 17.9 (C-5''), 63.1 (-OCH3). [4]

noranhyoicaritin (**15**): 1H-NMR (DMSO-d6, 600 MHz), 6.28 (1H, s, H-6), 8.03 (2H, d, J = 8.4 Hz, H-2', H-6'), 6.93 (2H, d, J = 9.0 Hz, H-3', H-5'), 3.42 (2H, d, J = 6.2 Hz, H-1''), 5.17 (1H, t, H-2''), 1.74 (3H, s, H-4''), 1.62 (3H, s, H-5''), 12.39 (1H, s, 5-OH). 13C-NMR (DMSO-d6, 150 MHz), 146.7 (C-2), 130.9 (C-3), 176.1 (C-4), 153.5 (C-5), 97.9 (C-6), 159.2 (C-7), 105.6 (C-8), 158.3 (C-9), 115.5 (C-10), 129.4 (C-1'), 122.8 (C-2',C-6'), 102.9 (C-3', C-5'), 21.2 (C-1''), 122.0 (C-2''), 25.5 (C-4''), 17.8 (C-5''). [3]

quercetin (**16**): 1H-NMR (DMSO-d6, 600 MHz), 6.18 (1H, d, J = 1.8 Hz, H-6), 6.40 (1H, d, J = 2.4 Hz, H-8), 7.67 (1H, d, J = 2.4 Hz, H-1'), 7.54 (1H, dd, J = 2.4, 8.4 Hz, H-5'), 6.87 (1H, d, J = 8.4 Hz, H-6'), 12.48 (1H, s, 5-OH). 13C-NMR (DMSO-d6, 150 MHz), 147.3 (C-2), 136.2 (C-3), 176.3 (C-4), 156.6 (C-5), 98.5 (C-6), 164.3 (C-7), 93.8 (C-8), 161.9 (C-9), 103.5 (C-10), 115.5 (C-2'), 145.5 (C-3'), 148.2 (C-4'), 116.1 (C-5'), 120.4 (C-6'). [8]

7,3'-di-O-methyl (**17**): 1H-NMR (DMSO-d6, 600 MHz), 8.72 (1H, s, H-2), 6.61 (1H, s, H-6), 6.77 (1H, s, H-8), 7.44 (1H, d, J = 1.8 Hz, H-2'), 7.35 (1H, dd, J = 1.8, 8.4 Hz, H-5'), 7.38 (1H, d, J = 8.4 Hz, H-6'), 2.92 (3H, s, 7-OCH3), 4.20 (3H, s, 3'-OCH3), 13.37 (1H, s, 5-OH). 13C-NMR (DMSO-d6, 150 MHz), 146.6 (C-2), 122.5 (C-3), 207.0 (C-4), 162.4 (C-5), 94.3 (C-6), 166.0 (C-7), 99.8 (C-8), 158.1 (C-9), 104.5 (C-10), 123.9 (C-1'), 112.4 (C-2'), 154.6 (C-3'), 158.1 (C-4'), 116.8 (C-5'), 120.2 (C-6'), 49.1 (7-OCH3), 56.1 (3'-OCH3). [9]

genistein (**18**): 1H-NMR (DMSO-d6, 600 MHz), 8.25 (1H, s, H-2), 6.24 (1H, s, H-6), 6.35 (1H, s, H-8), 7.34 (2H, d, J = 8.4 Hz, H-2', H-6'), 6.80 (1H, d, J = 8.4 Hz, H-3', H-5'), 12.93 (1H, s, 5-OH). 13C-NMR (DMSO-d6, 150 MHz), 154.2 (C-2), 121.7 (C-3), 180.5 (C-4), 162.1 (C-5), 99.4 (C-6), 164.9 (C-7), 94.1 (C-8), 158.0 (C-9), 104.7 (C-10), 122.7 (C-1'), 130.6 (C-2', C-6'), 115.4 (C-3', C-5'), 157.7 (C-4'). [8]

calycosin (**19**): 1H-NMR (DMSO-d6, 600 MHz), 8.34 (1H, s, H-2), 7.95 (1H, d, J = 9.0 Hz, H-5), 7.03 (1H, dd, J = 1.2, 8.4 Hz, H-6), 7.13 (1H, d, J = 1.2 Hz, H-8), 6.87 (1H, d, J = 1.8 Hz, H-2'), 6.93 (1H, dd, J = 1.8, 8.4 Hz, H-5'), 6.96 (1H, d, J = 7.8 Hz, H-6'), 3.15 (3H, s, 4'-OCH3). 13C-NMR (DMSO-d6, 150 MHz), 153.5 (C-2), 123.3 (C-3), 174.6 (C-4), 127.4 (C-5), 115.4 (C-6), 157.5 (C-7), 108.2 (C-8), 102.2 (C-10), 125.8 (C-1'), 109.5 (C-2'), 147.0 (C-3'), 147.1 (C-4'),116.5 (C-5'), 122.5 (C-6'). [8]

formononetin (**20**): 1H-NMR (DMSO-d6, 600 MHz), 8.32 (1H, s, H-2), 7.94 (1H, d, J = 8.4 Hz, H-5), 6.92 (1H, dd, J = 1.8, 9.0 Hz, H-6), 6.86 (1H, d, J = 1.8 Hz, H-8), 7.49 (2H, d, J = 8,4 Hz, H-2', H-6'), 6.97 (2H, d, J = 9.0 Hz, H-3', H-5'), 3.77 (3H, s, 4'-OCH3). 13C-NMR (DMSO-d6, 150 MHz), 153.2 (C-2), 123.2 (C-3), 174.8 (C-4), 127.3 (C-5), 116.6 (C-6), 159.0 (C-7), 102.1 (C-8), 157.6 (C-9), 113.7 (C-10), 124.4 (C-1'), 130.2 (C-2', C-6'), 115.2 (C-3', C-5'), 153.2 (C-4'), 55.2 (4'-OCH3). [10]

biochanin A (21): 1H-NMR (DMSO-d6, 600 MHz), 8.27 (1H, s, H-2), 6.21 (1H, s, H-6), 6.37 (1H, s, H-8), 7.35 (2H, d, J = 8,4 Hz, H-2', H-6'), 6.81 (2H, d, J = 8.4 Hz, H-3', H-5'), 3.16 (3H, s, 4'-OCH3), 12.95 (1H, s, 5-OH). 13C-NMR (DMSO-d6, 150 MHz), 154.2 (C-2), 130.3 (C-3), 180.2 (C-4), 157.7 (C-5), 93.8 (C-6), 164.3 (C-7), 99.1 (C-8), 161.8 (C-9), 104.5 (C-10), 122.4 (C-1'), 131.3 (C-2', C-6'), 115.9 (C-3', C-5'), 157.4 (C-4'), 48.7 (4'-OCH3). [10]

5,4'-dihydroxyflavone (22): 1H-NMR (DMSO-d6, 600 MHz), 6.24 (1H, s, H-3), 6.38 (1H, d, J = 8,4 Hz, H-6), 8.13 (1H, d, J = 9.0 Hz, H-7), 7.77 (1H, d, J = 9.0 Hz, H-8), 7.74 (2H, d, J = 9.6 Hz, H-2', H-6'), 6.84 (2H, d, J = 9.6 Hz, H-3', H-5'), 13.67 (1H, s, 5-OH). 13C-NMR (DMSO-d6, 150 MHz), 154.2 (C-2), 130.3 (C-3), 180.2 (C-4), 157.7 (C-5), 93.8 (C-6), 164.3 (C-7), 99.1 (C-8), 161.8 (C-9), 104.5 (C-10), 122.4 (C-1'), 131.3 (C-2', C-6'), 115.9 (C-3', C-5'), 157.4 (C-4'), 48.7 (4'-OCH3). [11]

luteolin (23): 1H-NMR (DMSO-d6, 600 MHz), 6.18 (1H, s, H-3), 7.42 (2H, m, H-6, H-8), 6.66 (1H, d, J = 1.8 Hz, H-2'), 6.89 (1H, dd, J = 1.8, 12.0 Hz, H-5'), 6.40 (1H, d, J = 12.0 Hz, H-6'). 13C-NMR (DMSO-d6, 150 MHz), 161.5 (C-2), 121.6 (C-3), 181.7 (C-4), 163.9 (C-5), 93.8 (C-6), 164.1 (C-7), 102.9 (C-8), 157.3 (C-9), 103.4 (C-10), 113.4 (C-2'), 149.7 (C-3'), 145.7 (C-4'), 116.0 (C-5'), 119.0 (C-6'). [12]

hydroxycoumarin (24): 1H-NMR (DMSO-d6, 600 MHz), 6.20 (1H, d, J = 9.6 Hz, H-3), 7.93 (1H, d, J = 9.6 Hz, H-4), 6.70 (1H, d, J = 2.4 Hz, H-5), 6.78 (1H, dd, J = 2.4, 8.4 Hz, H-6), 7.52 (1H, d, J = 8.4 Hz, H-8), 10.5 (1H, s, 7-OH). 13C-NMR (DMSO-d6, 150 MHz), 161.3 (C-2), 102.2 (C-3), 144,5 (C-4), 129.7 (C-5), 113.1 (C-6), 160.4 (C-7), 111.4 (C-8), 155.4 (C-9), 113.3 (C-10). [13]

7,8-dihydroxycoumarin (25): 1H-NMR (DMSO-d6, 600 MHz)，6.18 (1H, d, J = 9.6 Hz, H-3), 7.90 (1H, d, J = 9.6 Hz, H-4), 7.01 (1H, d, J = 8.4 Hz, H-5), 6.80 (1H, d, J = 8.4 Hz, H-6), 9.33 (1H, s, 7-OH), 10.09 (1H, s, 8-OH). 13C-NMR (DMSO-d6, 150 MHz), 160.9 (C-2), 112.5 (C-3), 145.6 (C-4), 119.3 (C-5), 112.9 (C-6), 150.1 (C-7), 132.6 (C-8), 145.6 (C-9), 111.7 (C-10). [13]

methoxysalicylic acid (26): 1H-NMR (DMSO-d6, 600 MHz), 6.21 (1H, d, J = 2.4 Hz, H-3), 6.28 (1H, dd, J = 2.4, 9.0 Hz, H-5), 7.59 (1H, d, J = 9.0 Hz, H-6), 3.42 (3H, s, -OCH3). 13C-NMR (DMSO-d6, 150 MHz), 172.1 (-COOH), 131.7 (C-1), 163.5 (C-2), 102.2 (C-5), 107.2 (C-6), 63.0 (-OCH3). [14]

b-resorcylic acid (27): 1H-NMR (DMSO-d6, 600 MHz), 6.24 (1H, d, J = 1.8 Hz, H-3), 6.31 (1H, dd, J = 2.4, 9.0 Hz, H-5), 7.50 (1H, d, J = 8.4 Hz, H-6). 13C-NMR (DMSO-d6, 150 MHz), 170.9 (-COOH), 132.0 (C-1), 163.7 (C-2), 102.2 (C-3), 163.4 (C-4), 104.9 (C-5), 107.8 (C-6). [14]

hydroxybenzoic acid (28): 1H-NMR (DMSO-d6, 600 MHz), 6.81 (2H, d, J = 8.4 Hz, H-2, H-6), 7.87 (2H, d, J = 8.4 Hz, H-3, H-5). 13C-NMR (DMSO-d6, 150 MHz), 167.9 (-COOH), 131.9 (C-1), 115.5 (C-2, C-6), 122.5 (C-3, C-5), 161.9 (C-4). [14]

2. DNA barcoding analysis

The dried crude materials were cleaned with 75% alcohol solution and ground into powder. The test was conducted according to our previous reports. Briefly, the dried crude materials were ground into powder. Total DNA was extracted from about 100 mg of the powder with the plant genomic DNA kit following the manufacturer’s instruction. The extracted DNA was dissolved in 30 μL of sterile water. The ITS sequence of S. flavescens sample was amplified by polymerase chain reaction (PCR). The primers for ITS sequence were designed as ITS-G-F (5′-GAAGG ATCAT TGTCG

ATGCC-3′) and ITS-G-R (5′-GCGTT CAAAG ACGCC TATTG G-3′). The PCR mixture (20 μL) contained template DNA 0.5 μL, forward primer (10 μM) 1.0 μL, reverse primer (10 μM) 1.0 μL, 5×TransStart KD Plus Buffer 4.0 μL, TransStart KD Plus DNA polymerase 0.4 μL, dNTP (2.5 mM) 0.4 μL, and ddH2O 12.7 μL. The PCR conditions were 94 °C for 3 min, then 35 cycles at 94 °C for 30 s, 56 °C for 30 s and 68 °C for 60 s, ending by incubating at 68 °C for 10 min. The PCR products were examined by 1.5% agarose gel electrophoresis before bi-directional DNA sequencing on a 3730XL sequencer (Applied Biosystems, USA). Sequence alignment and analysis of single nucleotide polymorphism on nucleotide chromatography were performed with DNAman (version 8.0, lynnon biosoft, USA) and BioEdit (version 7.0.0) software, respectively.

Table S1. ABTS free radical scavenging assay of *Sophora flavescens* compounds 1-28 (20 μg/mL).

**No.**	**compounds**	**ABTS**	**No.**	**compounds**	**ABTS**
**1**	kurarinol A	97.0% (1.8%)	**15**	noranhyoicaritin	97.0% (3.5%)
**2**	kurarinol B	94.0% (2.7%)	**16**	quercetin	99.0% (3.0%)
**3**	kushenol H	98.0% (2.3%)	**17**	7,3′-di-O-methyl	93.0% (2.0%)
**4**	kushenol L	90.0% (2.7%)	**18**	genistein	69.0% (1.4%)
**5**	kuraridinol	75.7% (1.5%)	**19**	calycosin	51.3% (2.4%)
**6**	kuraridine	98.0% (3.7%)	**20**	formononetin	23.3% (0.9%)
**7**	xanthohumol	87.9% (2.0%)	**21**	biochanin A	76.4% (1.3%)
**8**	isoliquiritigenin	67.8% (1.0%)	**22**	5,4′-dihydroxyflavone	76.3% (4.7%)
**9**	kushenol Q	92.0% (1.4%)	**23**	luteolin	96.0% (1.6%)
**10**	sophoraflavanone B	88.0% (2.1%)	**24**	7-hydroxycoumarin	16.8% (3.2%)
**11**	naringenin	55.4% (2.2%)	**25**	7,8-dihydroxycoumarin	97.8% (1.8%)
**12**	kurarinol	97.0% (2.8%)	**26**	4-methoxysalicylic acid	72.9% (3.0%)
**13**	kushenol A	81.5% (1.9%)	**27**	b-resorcylic acid	63.4% (1.4%)
**14**	sophoflavescenol	98.0% (1.4%)	**28**	4-hydroxybenzoic acid	2.2% (0.9%)

The values in the brackets refer to the standard deviations.

Table S2. DPPH free radical scavenging assay of *Sophora flavescens* compounds 1-28 (20 μg/mL).

**No.**	**compounds**	**DPPH**	**No.**	**compounds**	**DPPH**
**1**	kurarinol A	40.0% (4.9%)	**15**	noranhyoicaritin	95.0% (2.7%)
**2**	kurarinol B	42.3% (1.5%)	**16**	quercetin	95.0% (4.4%)
**3**	kushenol H	53.5% (0.4%)	**17**	7,3′-di-O-methyl	42.0% (1.4%)
**4**	kushenol L	37.1% (3.0%)	**18**	genistein	28.0% (6.2%)
**5**	kuraridinol	29.1% (8.7%)	**19**	calycosin	25.6% (1.1%)
**6**	kuraridine	41.9% (5.6%)	**20**	formononetin	20.2% (1.0%)
**7**	xanthohumol	34.7% (1.9%)	**21**	biochanin A	46.8% (1.8%)
**8**	isoliquiritigenin	37.1% (0.4%)	**22**	5,4′-dihydroxyflavone	34.6% (3.4%)
**9**	kushenol Q	30.4% (5.4%)	**23**	luteolin	94.0% (4.9%)
**10**	sophoraflavanone B	32.2% (1.9%)	**24**	7-hydroxycoumarin	25.2% (3.7%)
**11**	naringenin	24.5% (3.9%)	**25**	7,8-dihydroxycoumarin	94.0% (4.2%)
**12**	kurarinol	30.8% (1.9%)	**26**	4-methoxysalicylic acid	25.4% (4.9%)
**13**	kushenol A	25.6% (2.4%)	**27**	b-resorcylic acid	23.1% (2.5%)
**14**	sophoflavescenol	93.0% (2.0%)	**28**	4-hydroxybenzoic acid	20.4% (8.6%)

The values in the brackets refer to the standard deviations.

Table S3. PTIO free radical scavenging assay of *Sophora flavescens* compounds 1-28 (8 μg/mL).

No.	compounds	PTIO	No.	compounds	PTIO
**1**	kurarinol A	37.3% (3.4%)	**15**	noranhyoicaritin	68.0% (8.1%)
**2**	kurarinol B	41.0% (2.6%)	**16**	quercetin	62.0% (2.2%)
**3**	kushenol H	39.3% (1.7%）	**17**	7,3′-di-O-methyl	38.9% (2.4%)
**4**	kushenol L	39.4% (1.3%)	**18**	genistein	35.8% (2.0%)

**5**	kuraridinol	37.7% (1.8%)	**19**	calycosin	37.0% (0.7%)
**6**	kuraridine	40.6% (2.2%)	**20**	formononetin	36.2% (2.0%)
**7**	xanthohumol	40.3% (1.4%)	**21**	biochanin A	37.9% (0.6%)
**8**	isoliquiritigenin	39.9% (2.2%)	**22**	5,4′-dihydroxyflavone	39.4% (1.0%)
**9**	kushenol Q	36.7% (0.8%)	**23**	luteolin	43.3% (2.0%)
**10**	sophoraflavanone B	38.6% (1.3%)	**24**	7-hydroxycoumarin	36.6% (3.1%)
**11**	naringenin	37.8% (2.2%)	**25**	7,8-dihydroxycoumarin	46.3% (4.8%)
**12**	kurarinol	37.5% (1.7%)	**26**	4-methoxysalicylic acid	37.7% (1.6%)
**13**	kushenol A	35.6% (1.0%)	**27**	b-resorcylic acid	36.3% (1,9%)
**14**	sophoflavescenol	55.0% (9.4%)	**28**	4-hydroxybenzoic acid	37.3% (2.3%)

The values in the brackets refer to the standard deviations.

Table S4. HepG2 cell inhibitory activities of *Sophora flavescens* compounds 1-28 (10 μg/mL).

**No.**	**compounds**	**HepG2 cell**	**No.**	**compounds**	**HepG2 cell**
**1**	kurarinol A	84.1% (6.0%)	**15**	noranhyoicaritin	8.8% (1.2%)
**2**	kurarinol B	19.9% (15.8%)	**16**	quercetin	4.6% (4.5%)
**3**	kushenol H	1.9% (1.4%)	**17**	7,3′-di-O-methyl	8.7% (2.9%)
**4**	kushenol L	4.6% (1.3%)	**18**	genistein	1.0% (6.1%)
**5**	kuraridinol	5.6% (1.3%)	**19**	calycosin	4.3% (5.7%)
**6**	kuraridine	0.9% (3.7%)	**20**	formononetin	1.4% (2.7%)
**7**	xanthohumol	10.4% (3.2%)	**21**	biochanin A	7.1% (6.5%)
**8**	isoliquiritigenin	1.7% (1.7%)	**22**	5,4′-dihydroxyflavone	0.6% (3.3%)
**9**	kushenol Q	3.5% (1.7%)	**23**	luteolin	1.1% (4.1%)
**10**	sophoraflavanone B	9.3% (3.4%)	**24**	7-hydroxycoumarin	8.6% (5.5%）
**11**	naringenin	3.6% (10.6%)	**25**	7,8-dihydroxycoumarin	8.5% (8.0%)
**12**	kurarinol	0.9% (4.2%)	**26**	4-methoxysalicylic acid	1.5% (1.9%)
**13**	kushenol A	97.5% (0.1%)	**27**	b-resorcylic acid	1.9% (2.4%)
**14**	sophoflavescenol	11.4% (2.8%)	**28**	4-hydroxybenzoic acid	10.2% (4.0%)

The values in the brackets refer to the standard deviations.

Table S5. A549 cell inhibitory activities of *Sophora flavescens* compounds 1-28 (10 μg/mL).

**No.**	**compounds**	**A549 cell**	**No.**	**compounds**	**A549 cell**
**1**	kurarinol A	58.0% (6.5%)	**15**	noranhyoicaritin	13.4% (2.0%)
**2**	kurarinol B	17.2% (5.5%)	**16**	quercetin	12.1% (3.7%)
**3**	kushenol H	11.3% (0.7%)	**17**	7,3′-di-O-methyl	40.9% (2.0%)
**4**	kushenol L	8.2% (1.3%)	**18**	genistein	6.6% (2.0%)
**5**	kuraridinol	-0.2% (2.7%)	**19**	calycosin	6.4% (6.2%)
**6**	kuraridine	2.0% (2.2%)	**20**	formononetin	-6.2% (0.6%)
**7**	xanthohumol	16.4% (2.3%)	**21**	biochanin A	8.7% (1.9%)
**8**	isoliquiritigenin	-5.7% (0.6%)	**22**	5,4′-dihydroxyflavone	29.7% (0.8%)
**9**	kushenol Q	-10.9% (2.1%)	**23**	luteolin	41.3% (2.1%)
**10**	sophoraflavanone B	8.0% (0.4%)	**24**	7-hydroxycoumarin	21.4% (2.5%)
**11**	naringenin	14.7% (1.8%)	**25**	7,8-dihydroxycoumarin	32.4% (2.3%)
**12**	kurarinol	37.0% (0.3%)	**26**	4-methoxysalicylic acid	9.4% (4.0%)
**13**	kushenol A	17.6% (0.6%)	**27**	b-resorcylic acid	22.8% (2.2%)
**14**	sophoflavescenol	6.9% (1.7%)	**28**	4-hydroxybenzoic acid	15.5% (1.7%)

The values in the brackets refer to the standard deviations.

Table S6. MCF-7 cell inhibitory activities of *Sophora flavescens* compounds 1-28 (10 μg/mL).

**No.**	**compounds**	**MCF-7 cell**	**No.**	**compounds**	**MCF-7 cell**
**1**	kurarinol A	85.0% (2.0%)	**15**	noranhyoicaritin	5.8% (2.1%)
**2**	kurarinol B	10.3% (3.4%)	**16**	quercetin	11.9% (0.4%)
**3**	kushenol H	-4.5% (3.7%)	**17**	7,3′-di-O-methyl	5.3% (2.0%)
**4**	kushenol L	-0.7% (1.7%)	**18**	genistein	4.3% (1.6%)
**5**	kuraridinol	7.4% (3.1%)	**19**	calycosin	-0/9% (1.5%)
**6**	kuraridine	15.0% (2.2%)	**20**	formononetin	8.7% (2.8%)
**7**	xanthohumol	11.7% (4.2%)	**21**	biochanin A	1.0% (0.4%)

**8**	isoliquiritigenin	7.2% (3.3%)	**22**	5,4′-dihydroxyflavone	9.0% (1.4%)
**9**	kushenol Q	4.5% (5.9%)	**23**	luteolin	-4.7% (2.4%)
**10**	sophoraflavanone B	7.7% (2.0%)	**24**	7-hydroxycoumarin	5.7% (2.8%)
**11**	naringenin	0.7% (2.5%)	**25**	7,8-dihydroxycoumarin	4.8% (1.0%)
**12**	kurarinol	4.8% (1.8%)	**26**	4-methoxysalicylic acid	0.7% (1.4%)
**13**	kushenol A	11.5% (2.1%)	**27**	b-resorcylic acid	14.1% (2.0%)
**14**	sophoflavescenol	2.8% (4.0%)	**28**	4-hydroxybenzoic acid	1.1% (2.9%)

The values in the brackets refer to the standard deviations.

Table S7. LO2 cell inhibitory activities of *Sophora flavescens* compounds 1-28 (10 μg/mL).

No.	compounds	LO2 cell	No.	compounds	LO2 cell
**1**	kurarinol A	29.4% (1.6%)	**15**	noranhyoicaritin	7.7% (3.9%)
**2**	kurarinol B	27.9% (2.7%)	**16**	quercetin	9.8% (1.5%)
**3**	kushenol H	15.5% (3.6%)	**17**	7,3′-di-O-methyl	1.1% (7.7%)
**4**	kushenol L	3.2% (1.4%)	**18**	genistein	5.0% (0.6%)
**5**	kuraridinol	-7.2% (1.5%)	**19**	calycosin	16.6% (2.1%)
**6**	kuraridine	-0.2% (1.0%)	**20**	formononetin	5.8% (0.9%)
**7**	xanthohumol	13.9% (1.3%)	**21**	biochanin A	5.8% (1.3%)
**8**	isoliquiritigenin	-2.1% (2.6%)	**22**	5,4′-dihydroxyflavone	-4.6% (3.5%)
**9**	kushenol Q	5.3% (1.3%)	**23**	luteolin	19.8% (3.6%)
**10**	sophoraflavanone B	4.7% (1.9%)	**24**	7-hydroxycoumarin	-0.3% (4.2%)
**11**	naringenin	5.8% (2.0%)	**25**	7,8-dihydroxycoumarin	8.6% (2.7%)
**12**	kurarinol	11.4% (0.7%)	**26**	4-methoxysalicylic acid	-6.2% (0.9%)
**13**	kushenol A	26.8% (2.7%)	**27**	b-resorcylic acid	1.9% (3.7%)
**14**	sophoflavescenol	5.5% (3.0%)	**28**	4-hydroxybenzoic acid	2.8% (0.9%)

The values in the brackets refer to the standard deviations.

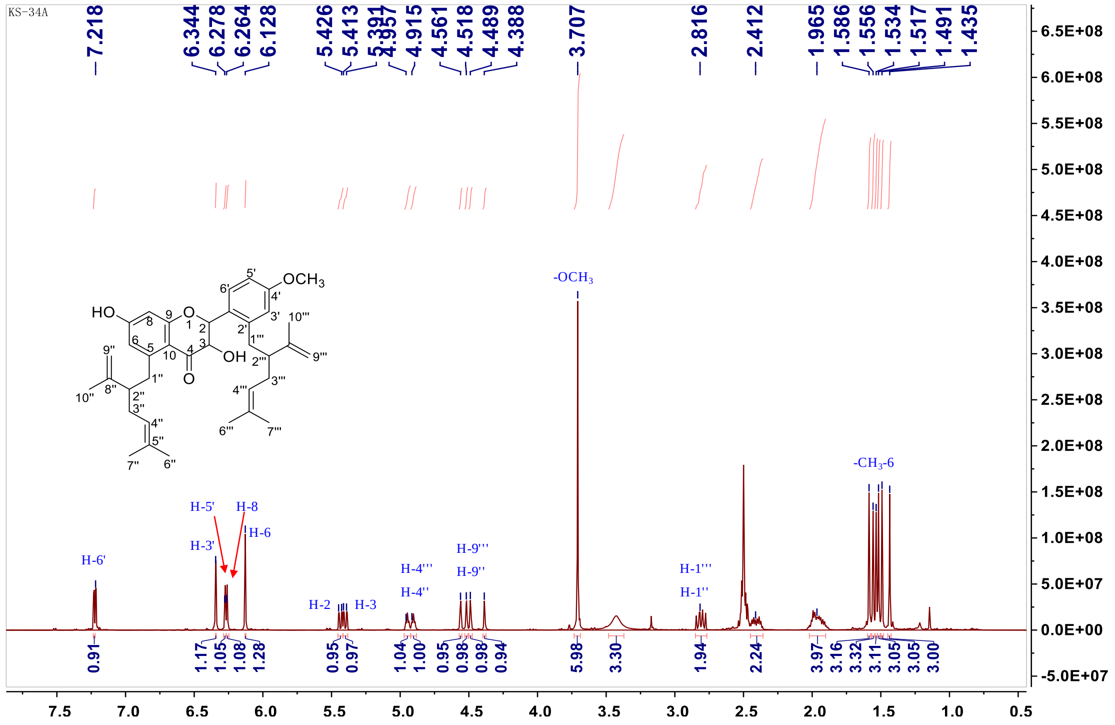

Figure S2. ^{1}H NMR spectrum of **1** in DMSO-d_6 (600 MHz).

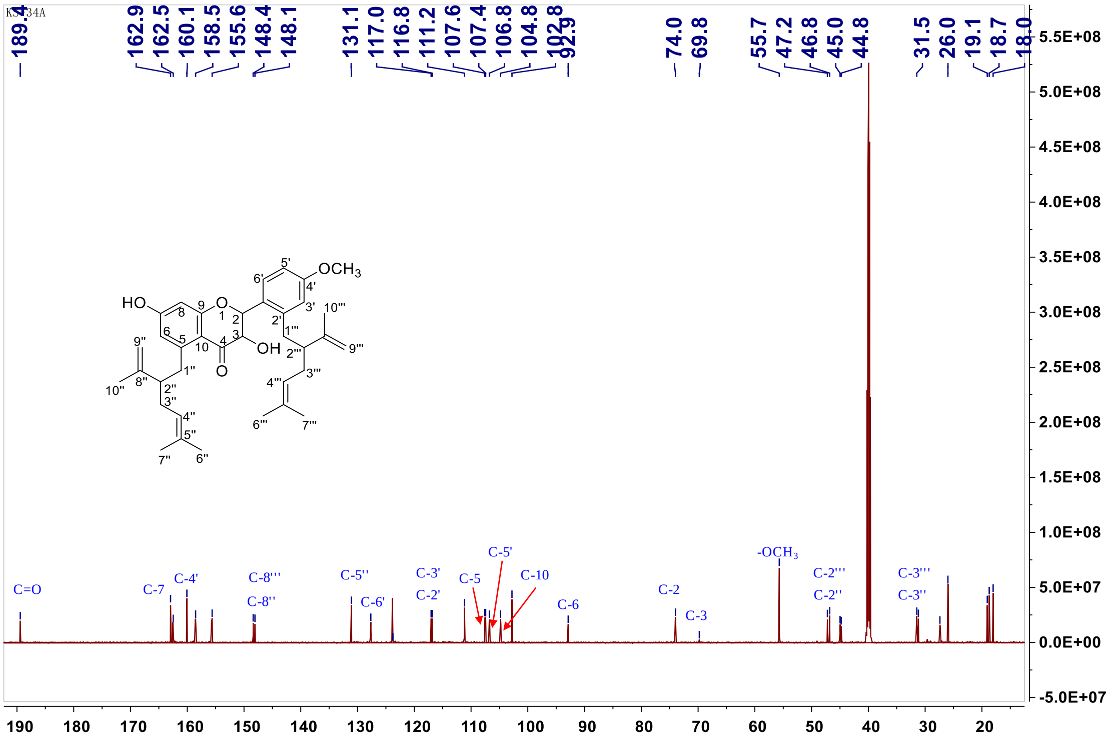

Figure S3. ^{13}C NMR spectrum of **1** in DMSO-*d*$_6$ (150 MHz).

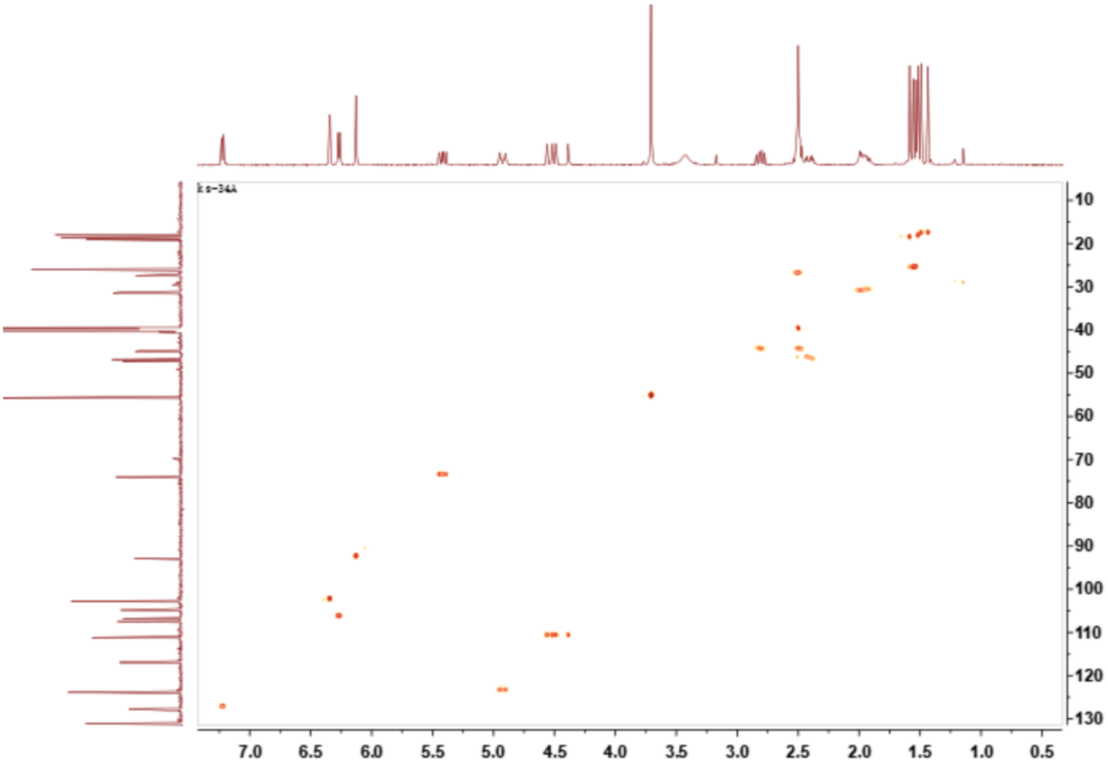

Figure S4. HSQC spectrum of **1** in DMSO-*d6* (600 MHz).

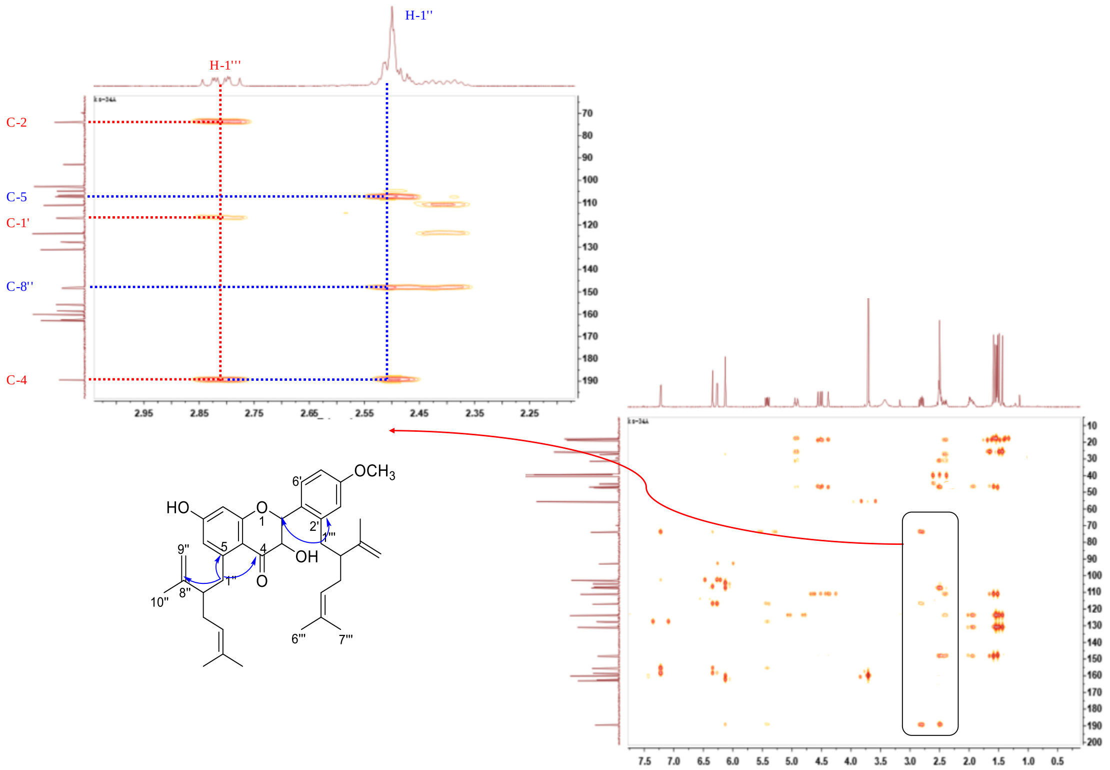

Figure S5. HMBC spectrum of **1** in DMSO-*d6* (600 MHz).

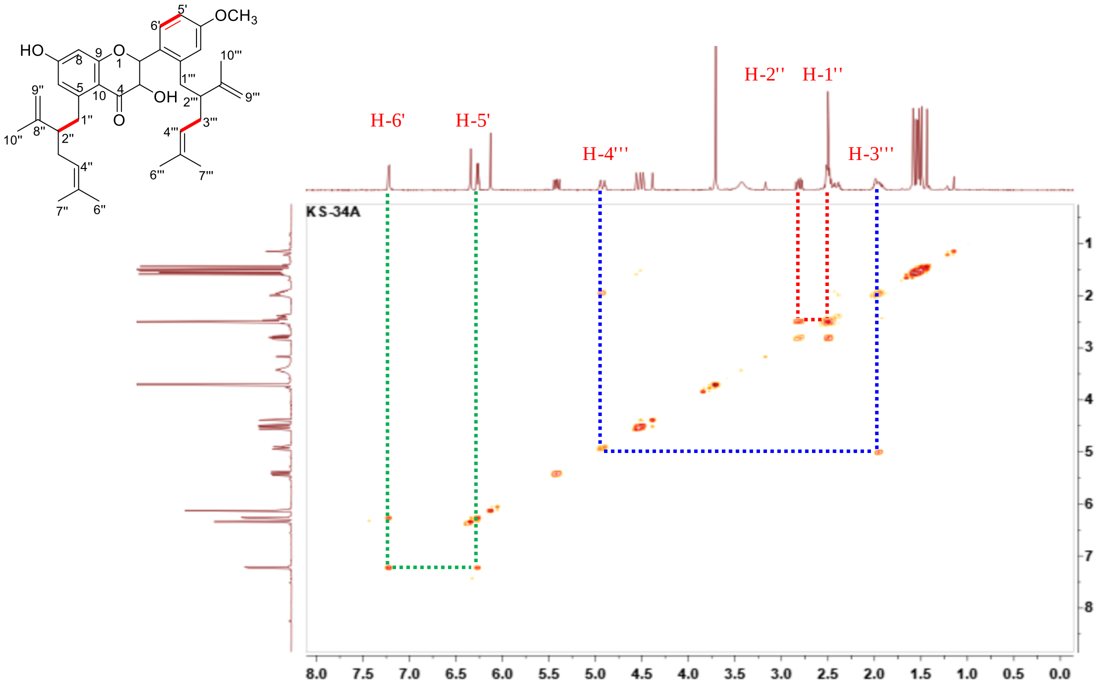

Figure S6. ^{1}H-^{1}H COSY spectrum of **1** in DMSO-*d6* (600 MHz).

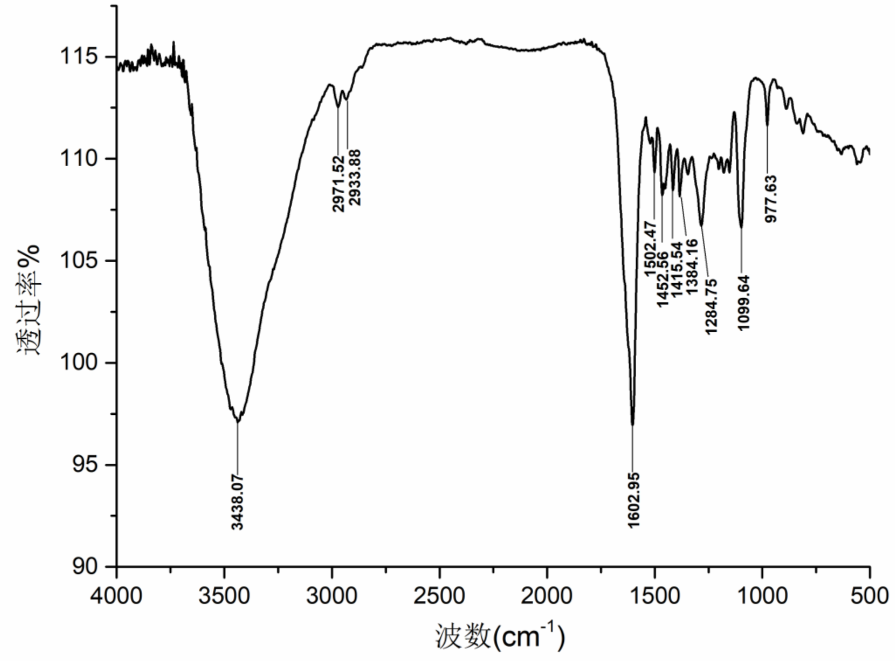

Figure S7. IR spectrum of **1**.

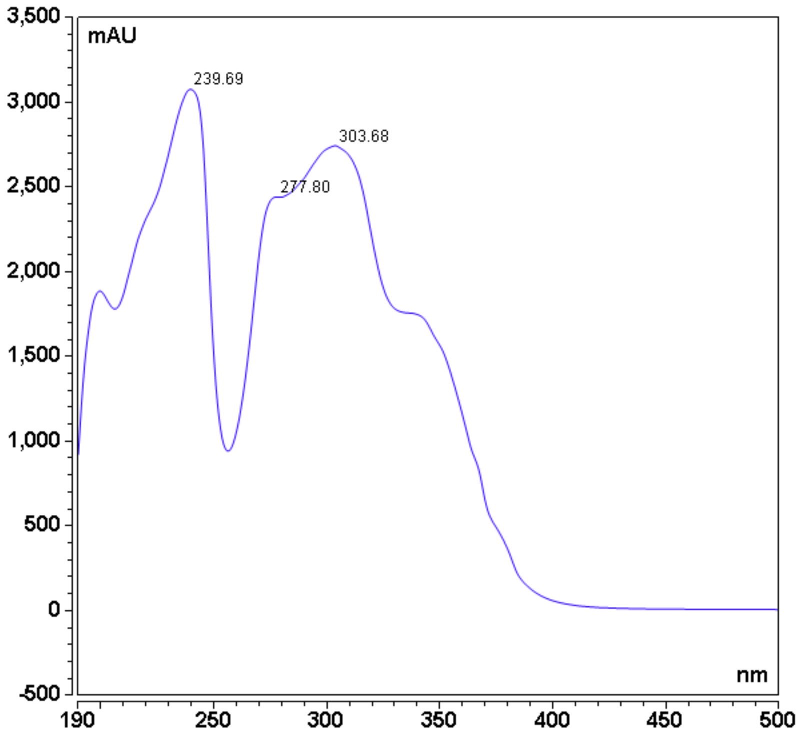

Figure S8. UV spectrum of **1**.

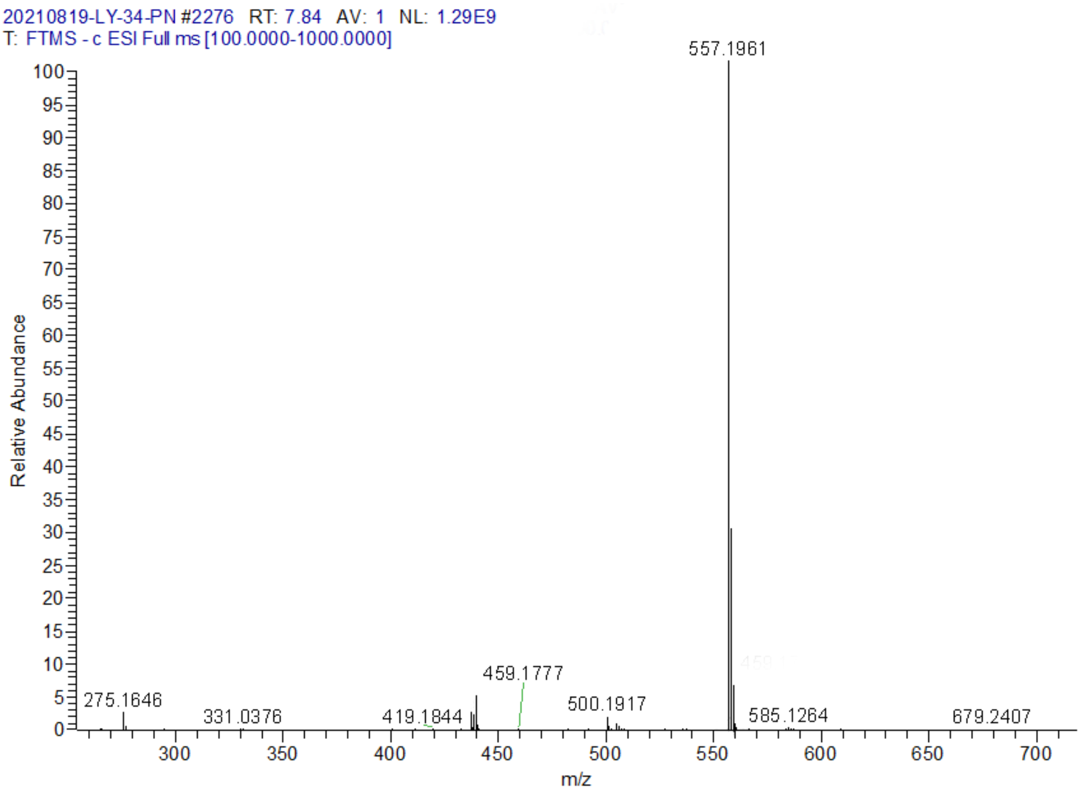

Figure S9. HRESIMS spectrum of **1**.

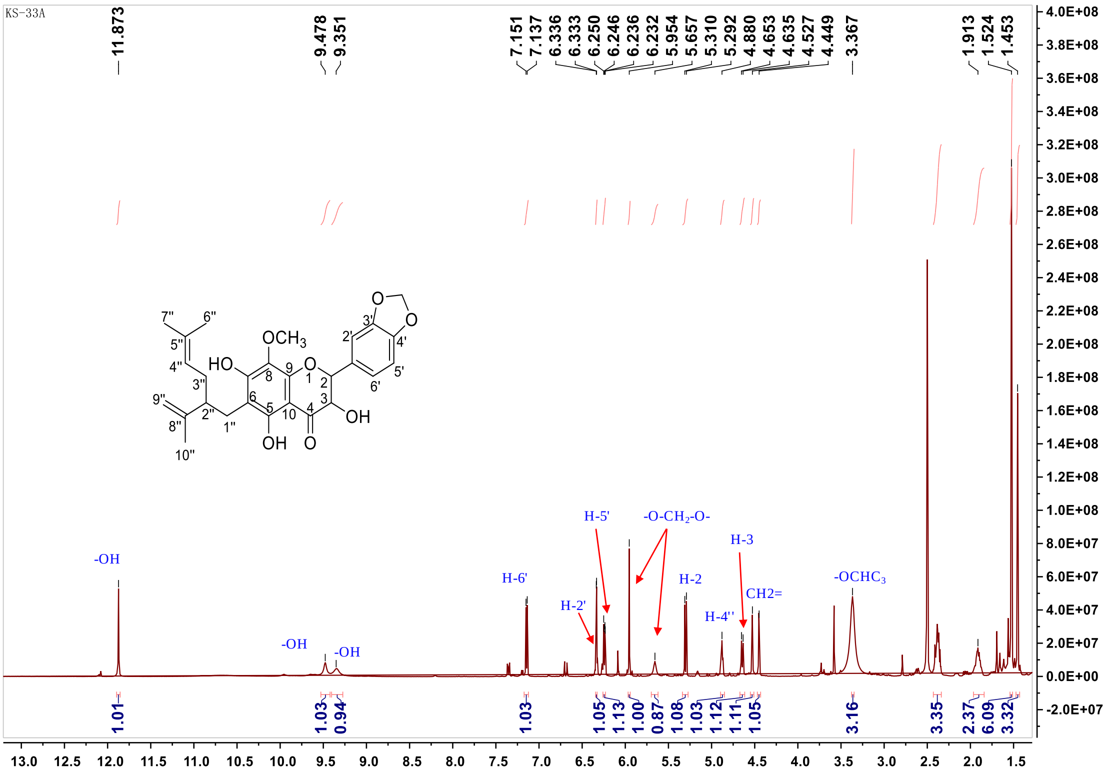

Figure S10. ^{1}H NMR spectrum of **2** in DMSO-d_6 (600 MHz).

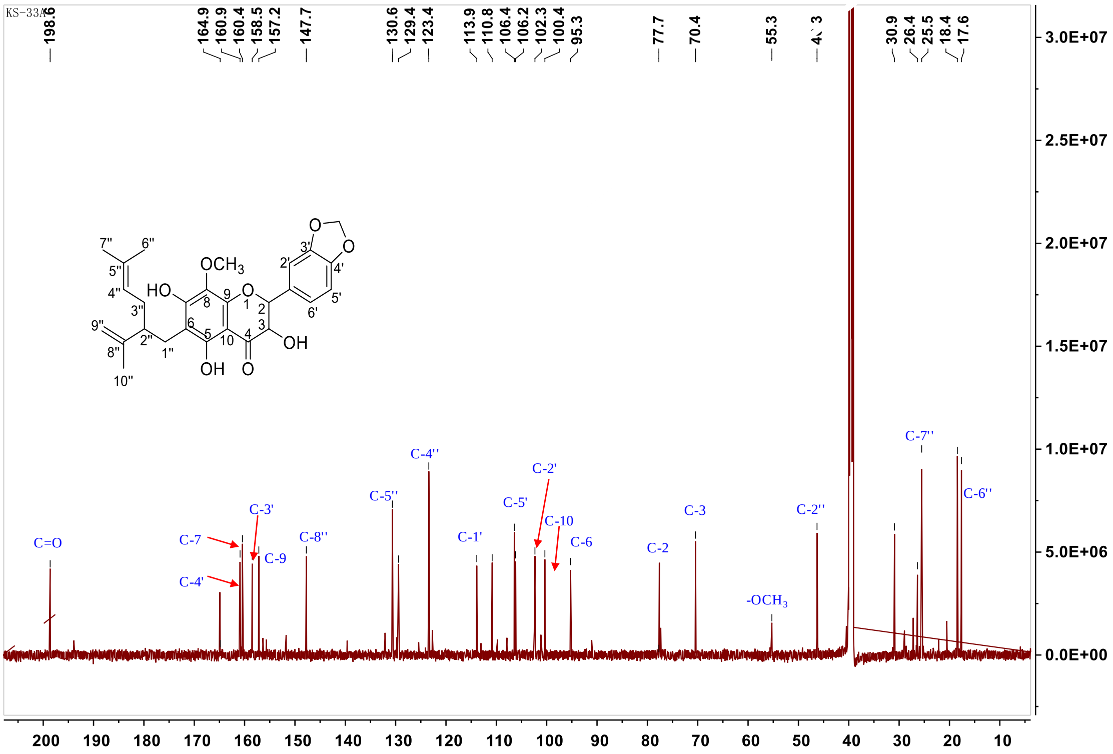

Figure S11. ^{13}C NMR spectrum of **2** in DMSO-*d6* (150 MHz).

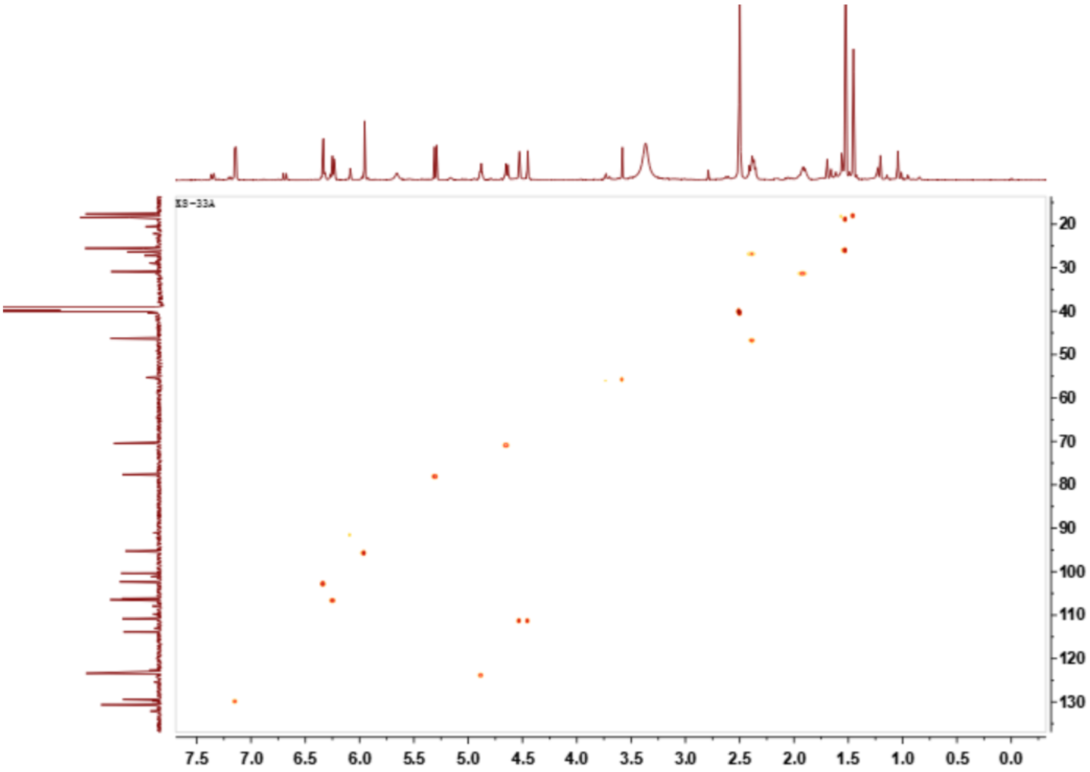

Figure S12. HSQC spectrum of **2** in DMSO-*d6* (600 MHz).

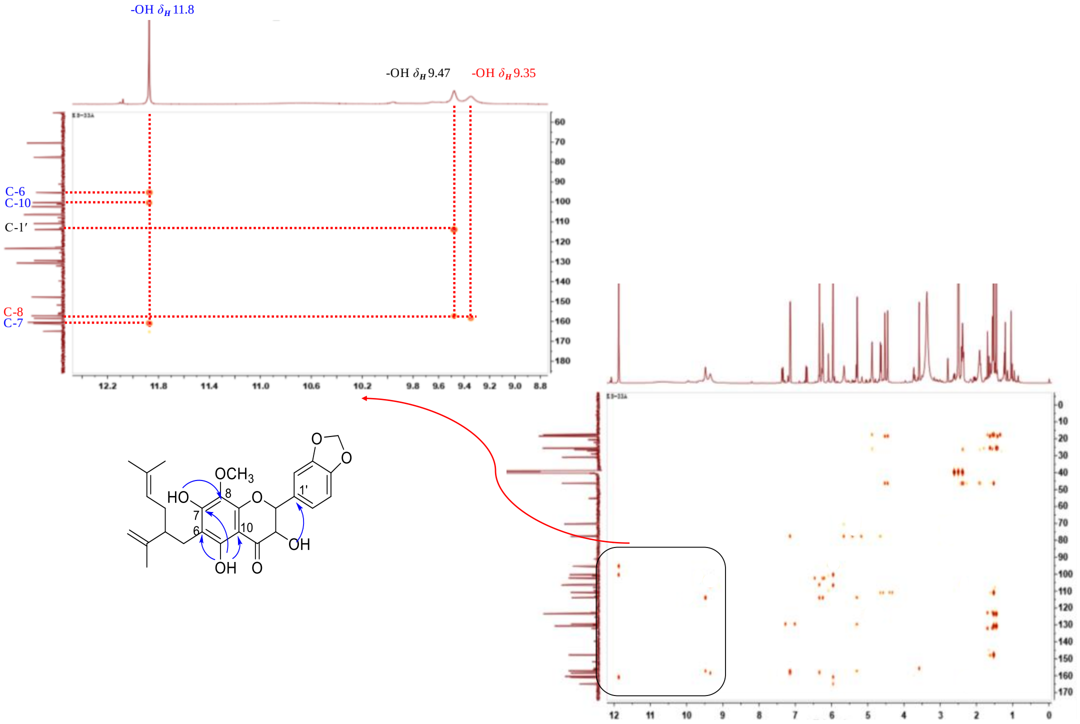

Figure S13. HMBC spectrum of **2** in DMSO-*d6* (600 MHz).

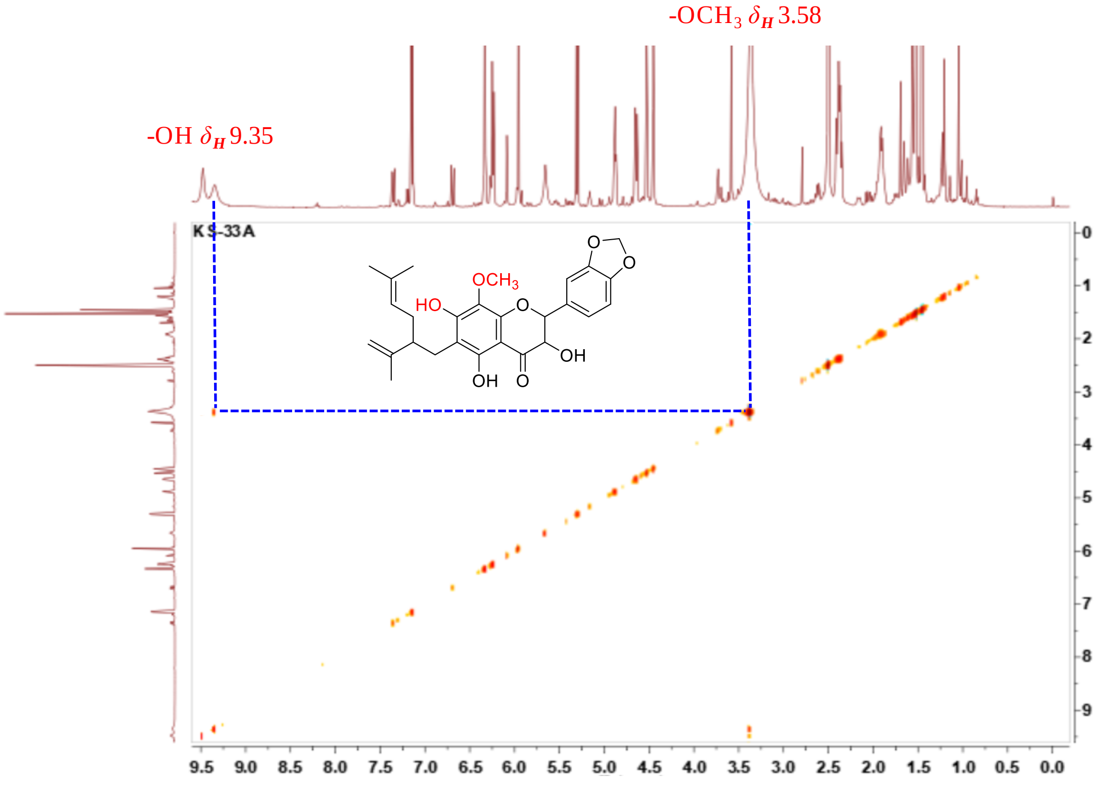

Figure S14. ^{1}H-^{1}H COSY spectrum of **2** in DMSO-*d6* (600 MHz).

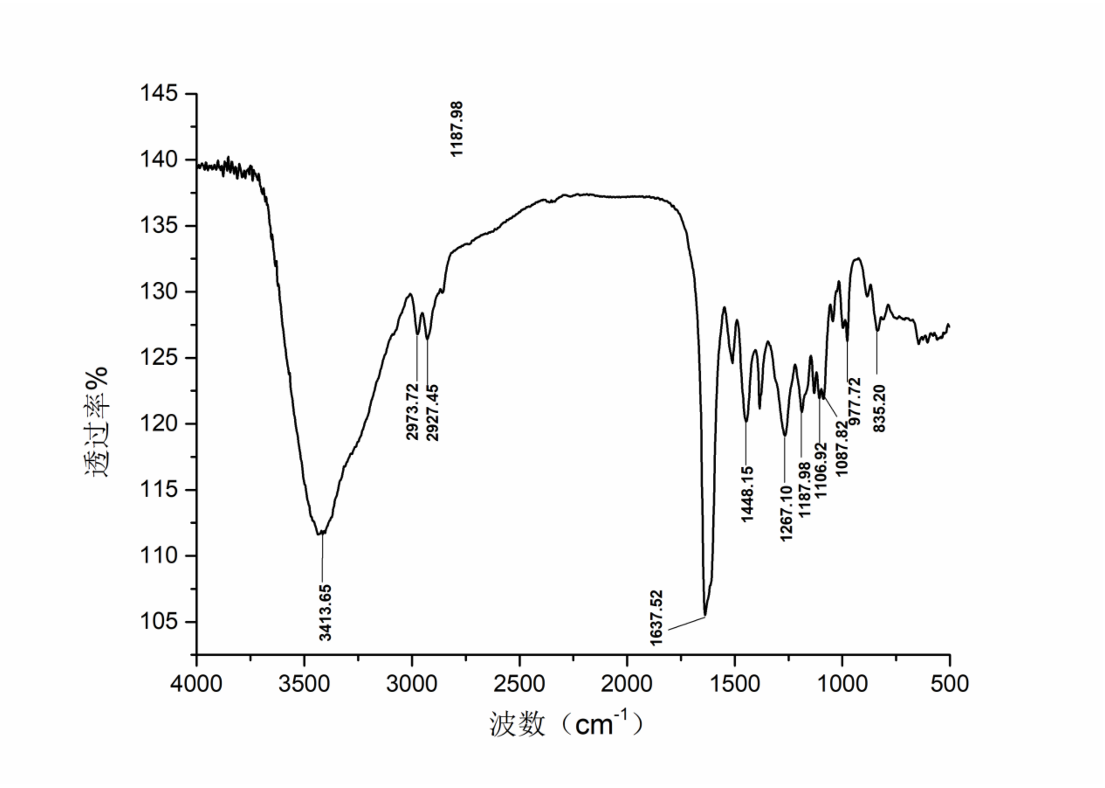

Figure S15. IR spectrum of **2**.

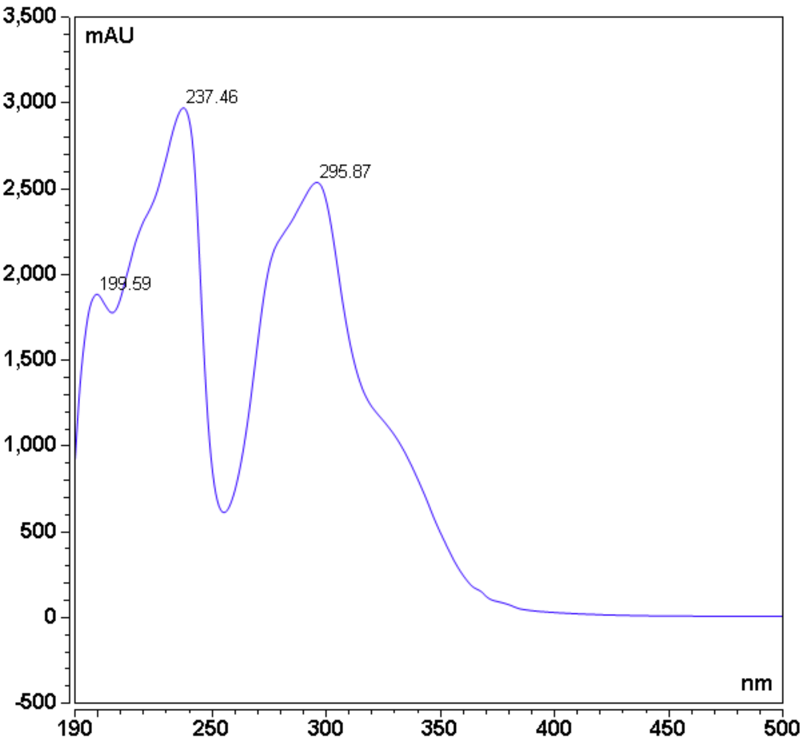

Figure S16. UV spectrum of **2**.

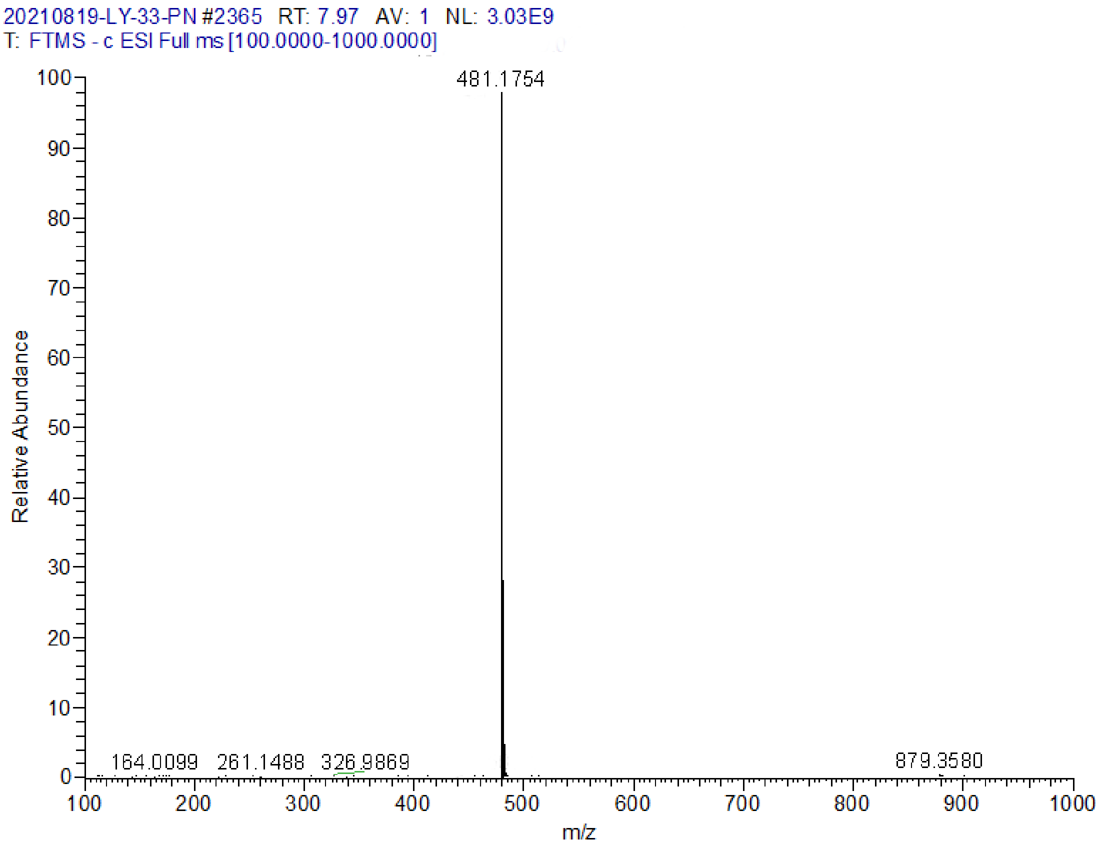

Figure S17. HRESIMS spectrum of **2**.

References

1. Kuroyanagi M, Arakawa T, Hirayama Y, Tatsuo H. Antibacterial and antiandrogen flavonoids from *Sophora flavescens*. Journal of Natural Products 1999; 62: 1595-1599
2. Ryu SY, Lee HS, Kim YK, Kim SH. Determination of isoprenyl and lavandulyl positions of flavonoids from *Sophora flavescens* by NMR experiment. Archives of Pharmacal Research 1997; 20: 491-495
3. Jung HA, Na YY, Kang SS, Kim YS, Choi JS. Inhibitory activities of prenylated flavonoids from *Sophora flavescens* against aldose reductase and generation of advanced glycation endproducts. J. Pharm. Pharmacol. 2008; 60: 1227-1236
4. Woo ER, Kwak JH, Kim HJ, Park H. A new prenylated flavonol from the roots of *Sophora flavescens*. J. Nat. Prod. 1998; 61: 1552-1554
5. Tamotsu S , Hiroshi N , Shoji S . A New: Isoflavone and Corresponding Isoflavanone of Licorice Root. Chem. Pharm. Bull. 1978; 26: 144-147
6. Lu Y, Irani NG, Grotewold E. Covalent attachment of the plant natural product naringenin to small glass and ceramic beads. Bmc Chemical Biology 2005; 5: 1-9
7. Li JW, Toshio M, Akira U, Masanori K, Tadataka N, Seigo F. Studies on the Constituents of *Sophora flavescens* AITON. II. Chemical & Pharmaceutical Bulletin 1985; 33: 3231-3236
8. Babiaka SB, Ntie-Kang F, Ndingkokhar B, Ndingkokhar B, Mbah JA, Sippl W, Yong JN. The Chemistry and Bioactivity of Southern African Flora II: Flavonoids, Quinones and Minor Compound Classes. ChemInform 2015; 46: 55704-55720
9. Herz W, Pethtel KD, Raulais D. Isoflavones, a sesquiterpene lactone-monoterpene adduct and other constituents of Gaillardia species. Phytochemistry 1991; 30: 1273-1279
10. Choi YH, Hong SS, Shin YS, Hwang BY, Park SY, Lee D. Phenolic compounds from *Pueraria lobata* protect PC12 cells against Aβ-induced toxicity. Archives of Pharmacal Research 2010; 33: 1651-1654
11. Pogodaeva NN, Medvedeva SA, Sukhov BG, Shishmareva TM. Hydrophobicity constants for several xanthones and flavones. Chemistry of Natural Compounds 2011; 47: 38-42
12. Wu SH, Nie FH, Chen QZ, Sun JJ. Electrocatalytic oxidation and nanomolar detection of hydrazine by luteolin electrodeposited at a multi-walled carbon nanotube and ionic liquid composite modified screen printed carbon electrode. Analytical Methods 2010; 2: 1729-1736
13. Kang KS, Ahn BZ. Antineoplastic Natural Products and the Analogues VIII Synthesis of some Coumarins and Their cytotoxic Activities on LI210 Cell. Arch. Pharm. Res. 1986; 9: 115-117
14. Gootjes J, Voorspuij AZ, Nauta WT. Investigation into the tuberculostatic activity of some aromatic hydroxy compounds. Antonie Van Leeuwenhoek 1953; 19: 237-245
